# Determinants of Pesticide Exposure in Occupational Studies: A Meta-Analysis

**DOI:** 10.3390/toxics11070623

**Published:** 2023-07-18

**Authors:** Christelle Oltramare, Zakia Mediouni, Yara Shoman, Nancy B. Hopf, Halshka Graczyk, Aurélie Berthet

**Affiliations:** 1 Center for Primary Care and Public Health (Unisanté), Department of Occupational and Environmental Health (DSTE), University of Lausanne, 1066 Epalinges-Lausanne, Switzerland; christelle.oltramare@unisante.ch (C.O.);; 2 International Labour Organization (ILO), 1211 Geneva, Switzerland

**Keywords:** determinant of exposure, pesticides, occupational exposure

## Abstract

Few epidemiological studies use exposure determinants specifically tailored to assess pesticide or plant protection product (PPP) exposures when assessing presumed association between occupational exposure and health outcomes among agricultural workers. This lack of exposure specificity could lead to results that fail to detect an association. It could be related to the lack of consensus on exposure assessment methods and the choice of exposure determinants. We conducted a meta-analysis following the PRISMA checklist to identify PPP exposure determinants used in occupational studies and identified exposure determinants that best characterized agricultural exposures to PPPs. Out of 1436 studies identified, 71 were included. The exposure determinants identified were active ingredients, chemical classes, types of PPP, crops, tasks, frequencies, duration, lifetime exposure days, and intensity-weighted exposure days. Only six over 17 associations between exposure determinants and health outcomes were found with moderate quality of evidence. Overall, epidemiological studies had difficulty defining relevant determinants to characterize PPP exposures for agricultural workers. We recommend that a standardized list of determinants for PPP exposures in occupational exposure studies should include information on formulations, intensity, duration, and frequency of PPP exposure. Harmonized data collection on exposure and health outcomes are required as well as standard units for each exposure determinant.

## 1. Introduction

Occupational risk assessment is an evaluation of the likelihood that an adverse health effect induced by an event, such as Plant Protection Product (PPP) exposure during agricultural tasks, would occur [1]. In these assessments, PPP exposure information is combined with the hazard that derives from the active ingredient. Coformulants in the PPP formulation are not considered. Agricultural workers performing PPP application-related tasks may be exposed to PPPs. PPP exposure depends on the type of activity (e.g., mixing, loading, applying, re-entering, harvesting), application method (e.g., backpack sprayer; broadcast from farm vehicle; air sprayer), use of personal protective equipment (PPE), and other parameters [2,3]. Depending on the route of entry of a given substance, i.e., via skin, ingestion or inhalation [4], its effects may vary. These parameters lead to heterogeneous exposure of workers, which makes risk assessment challenging.

Exposure to PPPs may cause adverse health effects such as neurological diseases, cancer, endocrine disorders, or reproductive disorders [5]. However, there are contrary conclusions regarding a presumed link between PPP exposure and health outcomes in epidemiological studies [6]. We believe that this inconsistency might be due to the lack of exposure assessment as well as disease ascertainment.

Exposure determinants are factors that predict possible exposures to a given substance [7]. Using a set of exposure determinants specifically developed for PPP exposures could refine exposure assessment and consequently, have a better chance of detecting a possible association between exposures and health outcomes in epidemiological studies. The standard method to assess occupational exposure group is the Similar Exposure Group (SEG) approach [8,9]. The SEG approach is widely used in industrial settings to assess possible inhalation exposure. However, it is not often used to characterize exposure for agricultural workers, since skin has always been a major route of exposures among agricultural workers. The validation of SEG is performed using air sampling. However, the main exposure route in agricultural workers is skin exposure (around 90% of exposure) followed by inhalation (around 10% of exposure) [4,10]. Moreover, workers are exposed to different formulations of products (the active ingredient and other ingredients like formulants) [11]. The different formulations can have a different kinetic effect.

Several epidemiological studies evaluating the potential link between exposure to PPPs and health outcomes are ambiguous and the reason might be that exposures to PPPs are rarely characterized [12]. Exposures were often not evaluated on exposure intensity, frequency, and duration. For example, an agricultural worker prepares and manually sprays the product over many days and another worker who does not need to prepare the product and whose sprayer is hooked up to the tractor that sprays for some hours would be classified in the same exposed group. This leads to misclassification and a bias toward the null [13]. Exposure determinants were used in a few epidemiological studies to assess exposures; however, there was no consensus on the choice of exposure determinants nor assessment methods (e.g., self-reported questionnaire, interview).

In this systematic review and meta-analysis, our objectives were to evaluate exposure determinants identified in the occupational epidemiological studies and recommend the most relevant based on their ability to characterize agricultural PPP exposures according to the quality of evidence of the association between exposure determinants and health outcomes. A focus is on the estimate of the association of the exposure determinant with the health outcome.

## 2. Methods

### 2.1. Protocol and Registration

This review protocol is available on the international database PROSPERO (Registration number: CRD42022293243). This systematic review was conducted following the Preferred Reporting Items for Systematic Reviews and Meta-Analysis (PRISMA) methodology [14] (see PRISMA checklist in the Supplementary Information).

### 2.2. Information Sources

We performed a systematic literature search in three databases: Web of Science (maintained by Clarivate analytics), MEDLINE via PubMed, and Scopus, in July 2022. In order to be exhaustive and not exclude changes over time, the search included all articles published from 1st January 1990 to 18th July 2022. Search strings are available in Supplementary Information (Supplementary text S1).

### 2.3. Eligibility Criteria

The inclusion criteria of studies for the systematic review were (a) the exposed population was agricultural workers; (b) the investigated health outcome was neurological, carcinogenic, mutagenic, reprotoxic (CMR), or endocrine disruption (included thyroid, diabetes); (c) ascribed exposure levels or measured exposures during PPP use; (d) English language; and (e) original research articles. Reviews, meta-analyses, letters to the editor and monographs were excluded. Likewise, studies that did not report health effects or focused on poisoning events (i.e., acute exposure) were excluded. All the papers reporting association using ever/never use categories were excluded.

Title, abstract, and keywords were used for a first-step screening of eligibility using the online tool RAYYAN [15]. After the first screening, articles were read to include only studies using exposure determinants for assessing PPP exposures. Two reviewers (C.O and A.B.) performed the screening and selection of articles independently. At least 10% of the articles were randomly selected and cross-checked. No automated tools were used, except the tool for titles and abstracts screening for keywords and exclusion criteria, which was performed using RAYYAN.

### 2.4. Data Extraction

Data was extracted from the articles that met the eligibility criteria after screening the abstract and the full text. An Excel file was used to extract relevant data from each publication and compile the following information: Authors’ names, Title, Publication year, Study design (e.g., epidemiologic, case control, cross-sectional, etc.), Health outcome type, Health outcome assessment type (e.g., clinical exams, registers, medical questionnaires, etc.), Study location (country), Crop type, Study period, Study population (n), and Active substances (Table S1, in the supplementary information). Exposure determinants were classified into three categories: sociodemographic data, general farming information, and PPP use practices. The relevance of this classification is discussed in the results’ section. All the results from this systematic review were analyzed for each health outcome independently. Risk estimates related to cancer were combined for all cancer sites in order to increase the statistical power of the results.

### 2.5. Quality Assessment and Grading

#### 2.5.1. Meta-Analysis

We grouped studies depending on the outcome measured such as cancer, neurotoxicity, or endocrine disruption (diabetes and thyroid disruption). We further divided these four main groups into subgroups based on exposure determinants (e.g., active ingredient, chemical class, etc.). We performed the meta-analysis on the reported ratio (hazard risk (HR), odds ratio (OR), or relative risk (RR)) of the associations between the level in the exposure determinants groups and health outcomes. For example, in one study [16], they reported that the higher users (intensity-weighted lifetime days) of the herbicide 2,4,5-T had an increased risk of renal cell carcinoma (RR = 2.9 (95% CI: 1.65–5.17)) compared to the never users. To facilitate comparison, we converted the reported ratios (HR, OR, or RR) from the included studies into the most commonly reported ratios (HR, OR, or RR) when there were variations. This computation was performed using the formula published in studies [17,18], which takes into account the sample size and the reference rate. We used a restricted maximization likelihood model with the study ID as a random effect. We reported the summary estimates and heterogeneity I^2^ for the overall meta-analysis and subgroup analyses. In the meta-analysis, a summary estimate was obtained by combining the individual effect estimates (in this review RR or OR) observed in the original included studies that make up the analysis [19]. The I^2^ statistic quantifies the amount of variation in a meta-analysis that can be attributed to differences between trials rather than random sampling error. Mathematically, I^2^ is calculated as I^2^ = τ2/(σ2 + τ2), where τ2 represents between-trial heterogeneity, σ2 represents the shared sampling error across trials, and σ2 + τ2 represent the overall variation in the meta-analysis. Typically, I^2^ is derived from the formula (Q − df)/Q × 100%, where Q is the statistic for Cochran’s homogeneity test and df is the degree of freedom (equal to the number of trials minus one). Higgins et al. investigated various approaches to determine 95% confidence intervals for the I^2^ estimate [20]. The threshold to interpret I^2^ can be misleading due to the several factors that influence the inconsistency. Above 75%, there is considerable heterogeneity [20]. The publication bias was assessed using funnel plots when there were at least 10 studies in a subgroup [20]. Sensitivity analysis was performed by eliminating one study at a time and assessing the effect of this elimination by comparing the summary estimates and the heterogeneity (I^2^) before and after the elimination of each study [21].

#### 2.5.2. Risk of Bias and Quality Assessment

We used the Joanna Briggs Institute (JBI) checklist for prevalence studies to assess the risk of bias in each article [22,23]. The JBI’s critical appraisal tools help to determine the reliability, relevance, and outcomes of published articles. This checklist provides observations on internal and external validities on the methodological quality of the studies. The overall risk of bias for each study was categorized as either high risk of bias (i.e., more than (and equal to) five criteria out of nine rated “unclear” or “missing”) or low risk of bias (i.e., fewer than five criteria out of nine rated “unclear” or “missing”). The risk of bias was completed for included articles by the lead author (CO), and a second reviewer (AB) independently evaluated 10% of the articles selected randomly for cross-checking.

#### 2.5.3. Grading and Overall Quality of Evidence

We graded the quality of evidence of the associations between exposure determinants and health outcomes following the Grading of Recommendations Assessment, Development and Evaluation (GRADE) approach [24]. Following the GRADE, the quality of evidence could be high, moderate, low, or very low. The overall quality of evidence was assessed for each subgroup by evaluating the risk of bias, inconsistency, indirectness, imprecision, and publication bias. Risk of bias being already assessed by JBI, we assessed the remaining criteria. Inconsistency describes how consistent the effects are across included studies. Indirectness compares differences in intervention (exposure in our case), outcome measurements, or population in the studies. For example, there were differences between the population of interest and those who participated in relevant studies. Our systematic review included only farmers who met our eligibility criteria; hence, the evidence regarding farmers was direct by definition. However, in some cases, there could be exceptions and differences between the population of interest and those in the included studies [25]. Imprecision assesses the risk of random errors. We systematically started the assessment by considering the quality of evidence as high and then downgraded it depending on the risk of bias, inconsistency, indirectness, imprecision, and publication bias.

## 3. Results

### 3.1. Selected Studies

**Figure 1:**
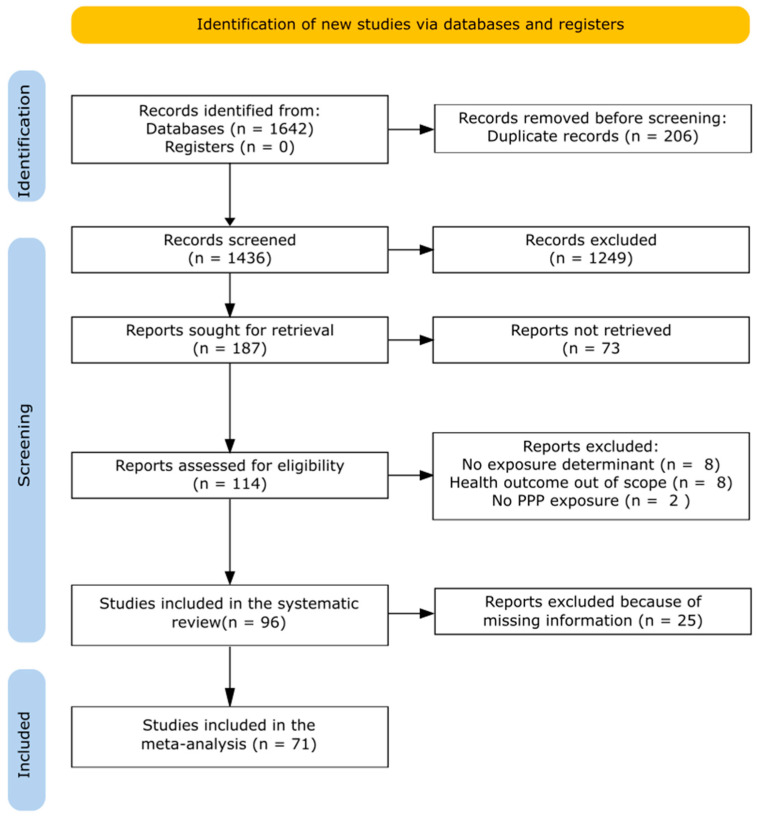
PRISMA 2020 flow chart of publication selection.

A total of 1642 records were identified with the literature search in three databases: Web of Science, PubMed, and Scopus (Figure 1). Among them, 206 were duplicates, leaving 1436 articles for the first screening based on titles and abstracts. Following the first screening, 364 articles were excluded because they were not original studies, 43 articles were excluded as they were not published in English and 842 articles were judged to be out of topic. Out of the remaining 187 original articles, 73 were excluded after the full-text screening and 18 were excluded for eligibility (Figure 1). Additionally, 25 studies were excluded from the meta-analysis because they did not report information (i.e., sampling size and population size) to transform the reported ratios into RR to allow comparison among studies (as described in Section 2.5.1). At the end, 71 studies were included in the meta-analysis (Supplementary information, Table S1).

Among the included studies in the systematic review, 37% were published in 2015 or later, 14% between 2010 and 2014, 39% between 2005 and 2009, and 10% between 2000 and 2004. There was no publications before 2000, although studies published after 1990 were included in the systematic review. Over 80% (84.5%) focused on a North American population, followed by a European (14.1%) and an Asian (1.4%) population. The included studies were mainly cohort studies (92%) and some case controls (8%). The majority of the studies (85%) focused on cancer, while endocrine disruption and neurotoxicity health effects made up 8 and 7% of the studies, respectively.

We could not provide a meta-analysis for the two endpoints of mutagenicity and reprotoxicity because the identified studies did not define the outcome in comparable ways. For example, some studies measured reprotoxicity as the number of miscarriages whereas other studies evaluated the sperm quality.

Health outcomes were mostly extracted from registries (82%) or from self-reported questionnaires (11%). Only a few studies integrated a medical doctor in the team to examine participants and diagnose or label their pathologies (3%) or administer psychometric tests to participants (3%). Even fewer studies used biomonitoring (1%).

Among the included studies, 6% were with a low risk of bias, 76% with a moderate risk of bias and 18% with a high risk of bias (Table S2). The low risk of bias articles listed on average several exposure determinants, and the health outcomes were mainly cancer. Most articles with moderate risk of bias did not include a representative sample of the population either because of convenience sampling or an inadequate response rate. Articles with a high risk of bias lacked detailed descriptions of the study subjects, suffered from a lack of information, control groups, or an association measurement.

The Table 1 listed the exposure determinants used to classify participants according to their PPP exposures in the 71 included studies. Twenty-five exposure determinants were identified. Each determinant was defined and assessed in a specific unit of measurement. Reported exposure determinants were very specific in some studies, such as which personal protective equipment (PPE) is used to perform a specific task or more general ones like spraying tasks (Yes or No). Some of the studies combined several exposure determinants to calculate an intensity level (IL) or a cumulative exposure index (CEI) [2]. These measurement tools were implemented initially in the American cohort Agricultural Health Study (AHS) to assess an overall quantitative long-term exposure [2]. The IL and CEI combined different exposure determinants listed in Table 1. A large number of studies considered sociodemographic information such as gender and age as exposure determinants.

### 3.2. Meta-Analysis Results

The meta-analysis was first performed for each of the four health outcomes (i.e., Cancer: 60 studies; Neurotoxicity: five studies; Endocrine disruptions: thyroid: three studies, diabetes: three studies). Since heterogeneity was high (I^2^ > 60%) [26], we then divided these disease groups into subgroups by exposure determinants (Table 2). The subgroup analysis provided results with reduced heterogeneity below 30% for the cancer for active ingredient, PPP type, task, frequency, duration, and intensity weighted exposure days as well as for endocrine disruption outcomes and active ingredient and frequency. In some cases, when only two to three studies were included in the subgroup analysis, the heterogeneity equals zero, which means that heterogeneity might not be important [20]. The overall heterogeneity in the subgroup analysis for neurotoxicity was considered moderate (I^2^ = 43–63%).

The quality of evidence was moderate for the association between two subgroups of exposure determinants with significant results: lifetime exposure days (HR = 0.93, 95%CI = 0.91–0.96, I^2^ = 40.67), intensity-weighted exposure days (HR = 0.96, 0.95–0.98, I^2^ = 15.57), and cancer. We found a low quality of evidence for the effect of “task” as an exposure determinant on protecting (HR < 1) from cancer (HR = 0.86, 0.71–0.99) in two studies with heterogeneity I^2^ = 0. The associations between active ingredient (HR = 1.01, 0.99–1.03), chemical class (HR = 1.05, 0.89–1.2), type of PPP (HR = 1.03, 0.93–1.1), crops (HR = 1.01, 0.98–1.03), frequency (HR = 0.68, 0.08–1.27), and duration (HR = 1.09, 0.99–1.19) with cancer were not significant. We found q low quality of evidence for the type of PPP harmful effect (OR > 1) associated with neurotoxicity (OR = 1.18, 1.11–1.25) in two studies with heterogeneity I^2^ = 63.44. However, there was no significant association between active ingredient and neurotoxicity (HR = 1.01, 0.98–1.05). There was a low quality of evidence for the association of active ingredient with diabetes (HR = 1.03, 1.01–1.06, I^2^ = 8.01) in two studies as well as with thyroid disruption (HR = 1.08, 1.05–1.11, I^2^ = 47.23) in two studies. We found a moderate quality of evidence for the harmful effect of longer duration and diabetes (OR = 1.19, 1.01–1.38) in two studies with heterogeneity I^2^ = 0. There was no significant association between the frequency and diabetes (HR = 0.94, 0.79–1.08). There was a moderate quality of evidence between harmful effects of intensity-weighted exposure days with thyroid disorder (HR = 1.09, 1.06–1.12) in three studies with heterogeneity I^2^ = 16.56 (Table 2). Overall, the indirectness had a negative impact and downgraded the quality of evidence for the associations of all subgroups of exposure determinants due to the difference in outcomes assessment, especially for the outcome of neurotoxicity.

#### 3.2.1. Effects of the PPP Exposure Determinants on the Health Outcome Association

Combining several exposure determinants may improve the results of the meta-analysis for the outcome of cancer specifically (Table 3). For example, the heterogeneity decreased for the association between active ingredient and cancer outcome, but the summary estimate was significant when restricting active ingredient with duration (Table 3). This estimate indicated harmful effects of a longer duration of exposure to the active ingredient in developing cancer among exposed farmers.

#### 3.2.2. Publication Bias

The results of publication bias for three groups of determinants (i.e., active ingredient, lifetime exposure days, and intensity-weighted exposure days) associated with cancer are presented in the supplementary material (Supplementary Materials Figures S1–S3). The funnel plots showed an asymmetry in the distribution of studies on both sides of the plots. Consequently, we considered there is evidence for publication bias based on the funnel plots.

## 4. Discussion

### 4.1. Main Findings

This systematic review identified 25 exposure determinants (Table 1) in 71 original studies published in the last 30 years. PPP exposure is not comparably assessed in epidemiological studies because they used different exposure determinants. This review highlighted the difficulty in characterizing exposures with specific PPP exposure determinants for agricultural workers. We included only nine exposure determinants in the meta-analysis as these were the only ones evaluated in a minimum of two studies. Two exposure determinants were exposure indices for an intensity level (IL): intensity-weighted exposure days and the cumulative exposure index (CEI) using lifetime exposure days [2]. They combined several exposure determinants. The IL- or CEI-exposure calculation provided a scale to estimate the PPP exposures. The difference between the IL and CEI is that the CEI takes into consideration the duration and frequency of application [27]. As exposure determinants, IL or CEI provided a moderate quality of evidence for their association with cancer and a moderate quality of evidence for their association with thyroid disruption. In our review, 60% of the included studies used a combination of exposure determinants (IL or CEI) to assess the risk of exposure. All these studies used data from the AHS cohort. This approach, using a combination of exposure determinants, could be a tool for epidemiological studies and should be recommended for assessing the overall risk associated with PPP practices. In several other studies, an extensive questionnaire completed by the agricultural workers was used to characterize PPP exposures but not all the data collected were used to assess exposure. Most of the exposure studies reported their results based on a dichotomous category (i.e., exposed and nonexposed). Although, some studies listed exposure determinants in their methodology to assess exposures, they did not necessarily analyze these data [28,29,30]. This emphasized that information requested of participants is not used for assessing PPP exposure even when available.

Out of nine determinants, only lifetime exposure days and intensity-level exposure days had a moderate quality of evidence and a statistically significant association with cancer. This could be explained by the fact that these two determinants combine several exposure determinants (i.e., mixing status, application method, and PPE use). Similarly, the meta-analysis showed that the combination of exposure determinants in the analysis decrease the heterogeneity of the results.

### 4.2. Results’ Interpretation

Socio-professional data are reported in the selected articles as exposure determinants although they are covariates or adjustment variables. They do not imply any particular effect on the exposure level among agricultural workers [31]. For example, the association between gender and health outcome among agricultural workers has not been evaluated in the literature due to the low number of women applying PPPs. In our meta-analysis, some studies (n = 4) used female gender as one of the exclusion criteria. Likewise, age used as a cumulative value to define exposure duration cannot be considered as a determinant of exposure.

Job title may also induce a bias in exposure determinant when used as a proxy of exposure [28,32]. This exposure misclassification groups people who are assumed to be comparably exposed to the substance of interest based on an individual characteristic, while exposure is an external one [33,34]. Similar conclusions were drawn when job title was used as an exposure assessment method such, as Job Exposure Matrices (JEMs), concerning PPP exposure [35]. Indeed, not all the tasks performed by an operator are considered in the JEMs, possibly leading to an underestimation. For example, few JEMs considered re-entry tasks even though these tasks are suspected of leading to high PPP exposures similar to spraying and mixing tasks [33,36]. However, JEMs are validated tools, more effective for assessing health risks related to occupational exposures than self-reported questionnaires [33] and help to cover a large population at a lower cost [37]. In addition, most of the occupational epidemiological studies evaluated PPP exposures using a self-reported questionnaire [38]. It may be perceived as the easiest and quickest approach to collect information on PPP exposures; however, response and recall bias are known to occur. The main advantage of self-reported questionnaires is that it is a simple way to collect extensive information or data from many participants. Nonetheless, answers may be subjective or invalid depending on the individual’s tendency to respond to questions. This response bias may affect the validity and the reliability of the collected data [39]. Moreover, there is a poor correlation between the estimates from crop-based job-exposure matrices (CEMs) and the self-reported exposure assessment [40]. This can lead to an underestimate of PPP exposure prevalence from self-reported questionnaires. These questionnaires evaluate a qualitative exposure whereas CEMs evaluate semiquantitative or quantitative exposures. In summary, the exposure assessment using proxy or self-reported questionnaire is not accurate and can lead to misclassification.

Biomonitoring, on the contrary, gives quantifiable internal exposure measurements [41]. The use of specific biomarkers and a suitable biological matrix (e.g., hair, urine, blood) are key elements to target exposure [42,43]. Biomonitoring measures the PPP active substance or its metabolites in the body following exposure. Biomonitoring results are the dose absorbed by the workers in a biological matrix independently of the route of exposure, absorption variation, and timing [44,45,46]. Biomonitoring may be a useful tool in epidemiological studies to interpret health-based biological results for short and mid-term effects [47,48]. Biomonitoring can associate the internal dose with exposure determinants. The most relevant exposure determinants can then be included within toxicokinetic models to predict exposures for a larger cohort without collecting samples. Furthermore, biomonitoring results are quantified exposure doses, while questionnaires are exposure estimates prone to response bias. Very few studies in this review (n = 3) used the biomonitoring approach. Furthermore, variation within the outcome measures make it difficult to compare between several original studies (indirectness assessed in Table 2). It is more difficult to link exposure biomarkers to chronic health outcomes. Nonetheless, questionnaires or sampling information are required to interpret biomonitoring data. A study with direct (biomonitoring) and indirect exposure assessment methods will most likely reduce exposure misclassification. Overall, molecular epidemiological studies should include both biomonitoring for quantification of present exposures and questionnaires to characterize past exposures. Some questionnaires should be added to assess the overall risk associated with PPP practices. For example, to build a combination of exposure determinants, such as IL and CEI. Correlation between the IL and CEI intensity scores and urinary biomarker concentrations were investigated in several studies leading to divergent results [13,49,50,51,52]. These findings lead to the similar conclusion than the meta-analysis that several exposure determinants are required to evaluate occupational exposures. Some findings highlighted missing information in the algorithm, which could explain possible misclassifications such as the PPP formula (i.e., powder, granular), the use of adjuvant, or the condition of spaying (i.e., orchard) [13,51,52].

The hazard of a PPP active ingredient is defined as the inherent property that can cause a health effect [53]. Few studies considered specific active ingredients of PPPs in their exposure assessment. The ones that did showed low or very low quality of evidence for active ingredient association with any health outcome. All studies included in this systematic review disregarded the chemical properties of the active ingredient (e.g., partitioning coefficient, volatility, etc.) as an exposure determinant. The hypothesis is that quantifying PPP exposure does not depend on the active ingredient in occupational exposure itself but rather on how it is used [54]. Moreover, agricultural workers are exposed to a cocktail of active ingredients repeatedly and over many years [55]. The mixture effects of PPPs on human health are not well-established yet [56]; however, the active ingredient remains an important variable to associate exposure to a health outcome. Furthermore, PPP producers and companies formulate their own mixtures of surfactants and active ingredients. Hence, each formulation has its specific trade name and composition. Trade names were not directly used as an exposure determinant in the included studies. Instead, they used trade names to categorize PPPs under an active ingredient or a type of PPP. However, the potential toxicity of surfactants used in PPP formulations might be higher than the active ingredient itself [57]. A recent systematic review emphasized that the toxicological hazards of the mixtures should not be underestimated [58]. It would be important to compare the toxicity between different PPP formulations including similar active ingredient and similar application practices. It might be challenging in epidemiological studies on chronic exposure due to changes of formulation over time. In summary, the active ingredient property itself is not sufficient to assess the exposure risk.

When selecting relevant exposure determinants, it is important to consider the major routes of exposure. Even though it is known that agricultural workers are mainly exposed via skin, none of the studies considered here included exposure determinants to assess specifically the skin exposure. Furthermore, in vitro studies have shown that skin permeation varies depending on the PPP formulation and the dose [11]. The American Health Study (AHS) included dermal exposure assessed as PPE status (e.g., long sleeves, gloves, and goggles.). The PPE status was assessed with a score in this study. Exposures were assessed using certain criteria and grouped into exposure levels that were given a score (1, 1.4, …). To take into account skin exposure, they used an algorithm to reduce the exposure level scores according to PPE status. However, this algorithm simplifies the use of PPE by applying the same reduction factor to all three tasks assessed (mixing, applying and repairing) [2]. Inadvertent ingestion is another poorly assessed route although it could influence the exposure assessment [59]. For example, agricultural workers may not clean the equipment after using PPPs. One typical example is the contamination of PPPs found on the tractor steering wheel [60,61]. They might not have access to water while working in the field and might have their break eating without washing their hands.

In short, to accurately determine PPP exposure, exposure determinants should include the main route of exposure depending on the task and the active ingredient as well as the formulation.

Finally, the exposure determinant definition should be discussed. According to Burstyn and Teschke [7], exposure determinants are used by occupational hygienists and can be applied in epidemiological investigations. Each exposure determinant should always be carefully described and defined with qualifiers of evaluation. As illustrated in Table 1, frequency of application is a good example of inconsistent definition of an exposure determinant. Some studies collected the number of application event per days/week/season [62,63,64,65], while others reported the date of the first PPP application [66,67]. The lack of standardized frequency measurement makes comparisons difficult. The maximum frequency of application determined by regulation could be used to model frequency. It determines how often a PPP can be sprayed per growing season. However, this assumption is based on the worst-case scenario and does not always reflect reality. From our point of view, the most accurate definition for PPP application frequency is the number of applications per day using the hour units. This includes the total period of time worker may be in contact with the PPPs, namely from the beginning to the end of work, regardless of PPE use and types of tasks.

### 4.3. Strengths and Limitations

This review was based on three of the largest scientific databases and included various keywords using different synonyms to identify relevant studies. Although 25 exposure determinants were listed in the studies, we only included those that were evaluated in a minimum of two studies. Conducting a systematic review of occupational epidemiology, particularly on pesticides, is challenging due to the inherent limitations of the original studies and the heterogeneity of available evidence. The first limitation was the missing information in the studies: 18% of the articles included in this systematic review were of high risk of bias due to missing detailed description of the study participants or appropriate statistical analysis. Further studies should address these issues by providing the information (i.e., open-source publication). Other limitations are the response and recall bias from self-reported questionnaires, the exposure assessment and/or health outcomes assessment that could lower the quality of a study. Risk of bias assessment and the GRADE tools provide to be useful to assess quality studies and therefore the review overall quality.

### 4.4. Implications of the Findings

This review emphasized a lack of exposure characterization in epidemiological studies. This leads to difficulties in interpreting or associating PPP exposures to health outcomes. A standardized list of exposure determinants for PPP occupational exposures would be of great interest to harmonize occupational studies as well as to increase relevance in protecting human health. Although many studies with valuable exposure data were available, they were not always used by the study authors. Instead, the participants are grouped into dichotomous groups (exposed vs. nonexposed) for comparison. Studies including groups with different exposure levels required a sufficiently high number of participants to obtain a statistically significant result. Data collected from different studies should be combined to improve PPP exposure characterizations. Open-source publications could facilitate better use of these data especially by creating a global registry framework as suggested for human biomonitoring [48]. Harmonized data collection on exposures and health outcomes are required. With a consensus from the scientific community, exposure determinants pertaining to PPP exposures should be listed, units harmonized, and ranked for relevance. To characterize the occupational PPP exposures appropriate data on intensity, duration, and frequency such as IL and CEI exposure calculation, frequency in hours, and amount of PPP applied, are necessary to scale and quantitatively estimate long-term exposures. Data on PPP formulations should be favored over data on active ingredients, because of the higher toxicity of some adjuvants added to formulations compared to the active ingredient itself [57]. PPP formulations combined with other parameters, such as dosage per surface and surface treated, will give a better estimate for PPP exposure. In future studies, biomonitoring could be an alternative to measure internal dose as it takes into account all sources of exposure (occupational as well as environmental exposures and uptake through food or other contaminants) [68]. Before collecting samples in biomonitoring, knowledge on toxicokinetic processes of the active ingredients should be documented to accurately interpret biomonitoring results (internal dose) and define PPP exposures (external dose). Ideally, biomonitoring should include exposure biomarkers and early-effects markers related to health outcomes. This approach would add to the existing health-based data and improve risk assessments. Finally, to reduce reporting bias, health outcome should be as much as possible diagnosed from a physician instead of extracted from self-reported questionnaire.

Overall, there is a need for a standardized list of exposure determinants for PPP exposures in occupational exposure studies. Cohorts may be used to carry out risk assessments, however, specific uniformed data allowing pooling studies are required. Therefore, we recommend defining a standardized list of exposure determinant to collect as initiated by Dosemeci [2].

## 5. Conclusions

This review highlighted the high variability in epidemiologic studies (e.g., indirectness) and the importance of collecting PPP exposure determinants. The most frequent exposure determinants are the lifetime exposure days and the intensity-weighted exposure days, and we found a moderate quality of evidence for their protective effects against cancer occurrence. A standardized list should at least include intensity, duration, and frequency of PPP exposure as well as formulations. Biomonitoring should also be added to assess PPP exposure and health outcomes when relevant in epidemiological studies because it takes into account dermal uptake which is the main route of exposure in agricultural workers. This could further support to assess the risk for user health, including agricultural worker, for PPP exposure.

## Figures and Tables

**Table 1:** Variable studied and their respective definition, unit of measurement and use in the intensity level (IL) or cumulative exposure intensity (CEI) calculation in the included studies on occupational exposure to PPPs.

Variable	Definition	Measure	Used in IL or CEI ^1^
Socio-professional information			
Age	Date of birth	Years	
Gender	Men/Women (M/W) or Male/Female (M/F)	M/W or M/F	
Job title	Employment status (e.g., Manager; Workers; Employee) or Self-reported status	Title	
General farming information			
Crop	Category of cultivated plants (e.g., cereals; fruits; vegetables)	Crop type	
Cultivated Surface	Land area used for crops	Acres/Hectare	
Task	Piece of work performed within the job (e.g., PPP spraying; harvesting; re-entering)	Category of job or task	
PPP use			
Active ingredient	Biological active chemical compound	Chemical name	
Chemical class	Group of compounds with similar features (i.e., organophosphates, pyrethroids, carbamates, …)	Chemical class	
Type of PPP	Target pest category of PPP (e.g., fungicide; herbicide; insecticide)	Classification	X
PPP license	Obtained date of permit to apply PPP	years	
Read instruction	Operator read the use instruction provided on the label (e.g., dilution; personal protective equipment; mixing)	yes/no	
Mixing/loading	Operator prepared the PPP solution and filled the spraying tank	yes/no	X Detailed
Application method	Spraying tank type (e.g., backpack sprayer; broadcast from farm vehicle; air sprayer)	Categories	X Detailed
Tank volume	Amount of PPP solution contained in a tank	Gallon: liter (3.8:1)	
Cleaning	Operator washed the application material	yes/no	Detailed
Repair	Operator repaired engine	yes/no	X
Re-entry	Operator entered in field following treatment	yes/no	
Duration	Amount of time spent on application	hours or days	
Frequency	Number of years an operator applied PPP or Number of days per year an operator applied PPP or Number of months an operator applied PPP per cropping season or Number of application events per day/week/season or Number of application events over the last 12 months or Date of first PPP application	year number number number number date	
Percentage of treated area	Percentage of cultivated surface treated by an operator	percentage	
PPE use	Use of PPE while performing PPP tasks and/or Use of PPE while cleaning and/or Use of PPE after a PPP spill and/or Duration of use before changing gloves	yes/no yes/no yes/no hours	X Detailed Detailed Detailed
Personal hygiene	Operator washed their hands after PPP application	yes/no	Detailed

PPE: Personal protective equipment; PPP: Plant protection product. ^1^ Variable used in the calculation of the IL (Intensity Level) or CEI (Cumulative Exposure Index) [2]. “X” means that the variable is taken in the calculation of IL or CEI, and “detailed” means that the variable is taken in the detailed calculation of IL or CEI.

**Table 2:** Summary of the results of meta-analysis of the association between exposure determinants and four outcomes with the quality of evidence grouped per health outcome. The overall quality of evidence is based on the GRADE approach, taking into account the risk of bias, inconsistency, impression, and indirectness. The reporting publication bias was not assessed for groups with fewer than 10 studies. (Na: not applicable.).

Exposure Determinant Sorted per Health Outcome	Number of Studies ^1^	Heterogeneity “I^2^ Estimate”	Summary Estimate of the Association with Health Outcome	Effect	95% Confidence Interval	Risk of Bias	Inconsistency	Imprecision	Indirectness	Publication Bias ^2^	Overall Quality of Evidence ^3^
Cancer	60										
Active Ingredient	14	17.17	1.009	Harmful	0.992–1.027	Moderate	No	Yes	No	Yes	Very low
Chemical class	5	60.94	1.053	Harmful	0.898–1.208	High	No	No	No	Na	Moderate
Type of PPP	6	22.96	1.033	Harmful	0.936–1.13	Moderate	No	Yes	No	Na	Low
Crops	9	44.25	1.006	Harmful	0.984–1.028	Moderate	No	No	No	Na	Moderate
Task	2	0	0.856	Protective	0.715–0.998	Moderate	No	Yes	No	Na	Low
Frequency	2	0	0.68	Protective	0.085–1.275	Moderate	No	Yes	No	Na	Low
Duration	3	0	1.096	Harmful	0.993–1.198	Moderate	No	Yes	No	Na	Low
Lifetime exposure days	28	40.67	0.932	Protective	0.906–0.959	Low	No	No	No	Yes	Moderate
Intensity-weighted exposure days	31	15.57	0.964	Protective	0.947–0.982	Low	No	No	No	Yes	Moderate
Neurotoxicity	5										
Active Ingredient	3	43.39	1.014	Harmful	0.979–1.050	High	No	No	Yes	Na	Low
Type of PPP	2	63.44	1.181	Harmful	1.109–1.254	High	No	No	Yes	Na	Low
Duration	2	0	1.353	Harmful	1.160–1.547	High	No	No	Yes	Na	Low
Endocrine disruptor— Diabetes	3										
Active Ingredient	2	8.01	1.034	Harmful	1.010–1.057	High	Yes	No	No	Na	Low
Duration	2	0	1.192	Harmful	1.006–1.377	High	No	No	No	Na	Moderate
Frequency	2	13.04	0.940		0.796–1.084	High	No	No	Yes	Na	Low
Endocrine disruptor— Thyroid	3										
Active Ingredient	2	47.23	1.078	Harmful	1.047–1.110	Moderate	Yes	No	No	Na	Low
Intensity-weighted exposure days	3	16.56	1.090	Harmful	1.061–1.120	Moderate	No	No	Yes	Na	Moderate

^1^ Number of studies grouped by heath outcome and determinant of exposure. The quality of evidence was assessed when at least two studies had similar determinant of a health outcome. ^2^ Funnel plots is available in the Supplementary information: Figures S1–S3. ^3^ Based on the GRADE, which takes into account the risk of bias, inconsistency, indirectness, imprecision, and publication bias of all studies for a given predictor.

**Table 3:** Results of the meta-analysis of the association between exposure determinants and cancer when restricting some exposure determinants with another exposure determinants. The significant results are highlighted in italics.

Cumulative Exposure Determinant Sorted per Health Outcome	Number of Studies	Heterogeneity “I^2^ Estimate”	Summary Estimate of the Association with Health Outcome	95% Confidence Interval
Cancer				
Chemical class + Duration	2	0	1.285	0.985; 1.046
Type of PPP + Duration	3	0	1.039	0.996; 1.042
Crops + Duration	4	3.06	1.016	0.959; 1.120
*Active ingredient + Duration*	*11*	*11.88*	*1.019*	*1.153; 1.418*

## Data Availability

No new data were created in this study. Data sharing is not applicable to this article. The articles included in the meta-analysis are listed in Table S1.

## Supplementary Materials

The following supporting information can be downloaded at: https://www.mdpi.com/article/10.3390/toxics11070623/s1, PRISMA 2020 Checklist; Text S1: String research keywords; Figure S1: Funnel plot on association between active ingredient and cancer for the evaluation of the publication bias; Figure S2: Funnel plot on association between lifetime exposure days and cancer for the evaluation of the publication bias; Figure S3: Funnel plot on association between Intensity-weighted exposure days and cancer for the evaluation of the publication bias; Table S1: 71 Articles included in the meta-analysis with additional data extracted; Table S2: Overall risk of bias assessment following the Joanna Briggs Institute (JBI) checklist for prevalence studies [16,62,64,65,69,70,71,72,73,74,75,76,77,78,79,80,81,82,83,84,85,86,87,88,89,90,91,92,93,94,95,96,97,98,99,100,101,102,103,104,105,106,107,108,109,110,111,112,113,114,115,116,117,118,119,120,121,122,123,124,125,126,127,128,129,130,131,132,133,134,135].
