# Supplementary material for: Determinants of Pesticide Exposure in Occupational Studies: A Meta-Analysis
Source: Toxics. 2023 Jul 18;11(7):623. doi: 10.3390/toxics11070623 (PMC10386710; doi:10.3390/toxics11070623)
Supplement: Supplementary file 1 [file toxics-11-00623-s001.zip › toxics-2475650-supplementary.pdf]

toxics

Supplementary information

Determinants of Pesticide Exposure in Occupational Studies: A Meta-Analysis

Christelle Oltramare[1], Zakia Mediouni[1], Yara Shoman[1], Nancy B. Hopf[1], Halshka Graczyk[2], Aurélie Berthet [1,*]

[1] Center for Primary Care and Public Health (Unisanté), Department of occupational and environmental health (DSTE), University of Lausanne, CH-1066 Epalinges-Lausanne, Switzerland
[2] International Labour Organization (ILO), 1211 Geneva, Switzerland
* Correspondence: aurelie.berthet@unisante.ch

Text S1: String research keywords

The search keywords were "pesticid* OR fungicid* OR insceticid* OR herbicid*"; "agricultural exposure* OR "agricultural health" OR agricultural work* OR vineyard*"; "occupational diseases OR occupational exposure OR occupational medicine OR occupational risk OR (industry AND mortality) OR occupational group* OR work-related OR working environment"; "cohort OR "case study" OR "cross sectional" ".

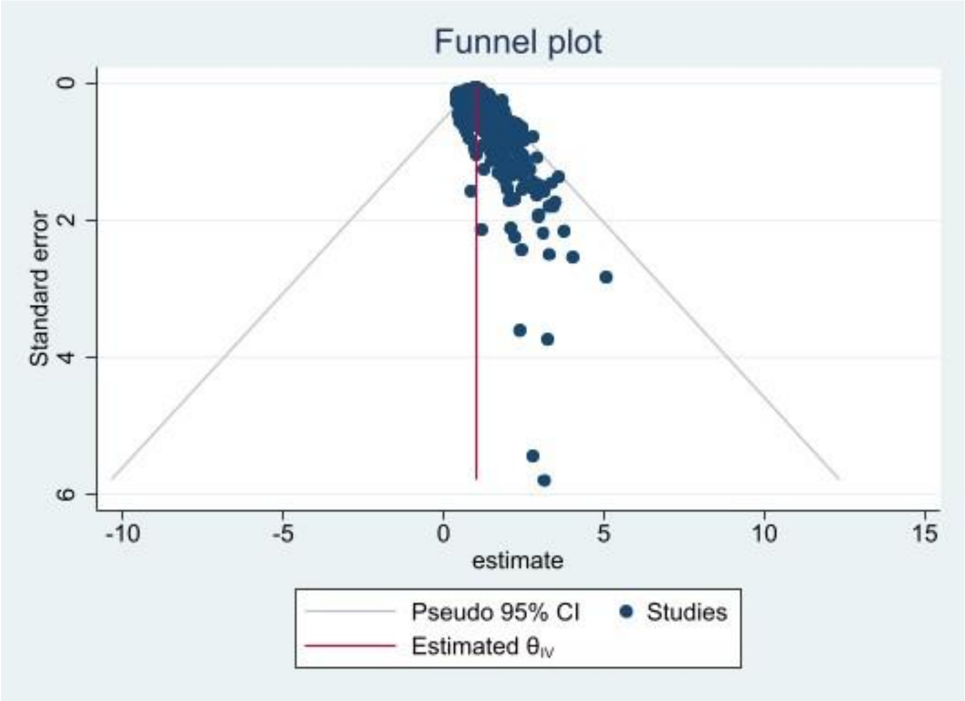

Figure S1. Funnel plot on association between *active ingredient* and *cancer* for the evaluation of the publication bias.

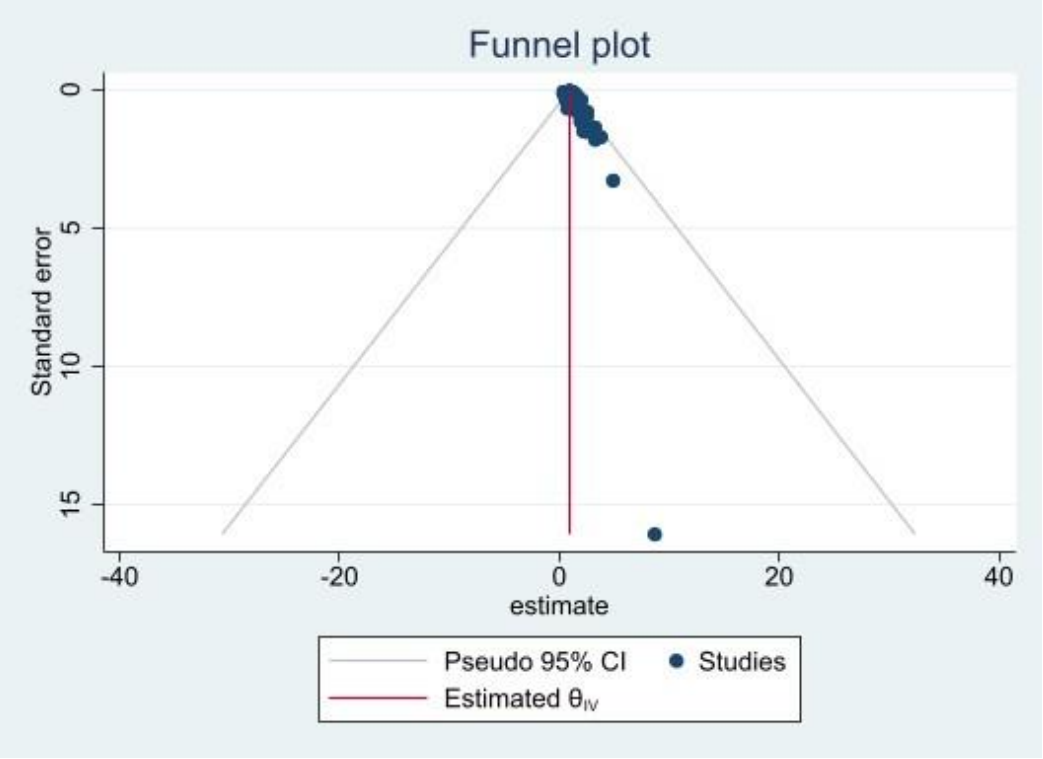

Figure S2. Funnel plot on association between *lifetime exposure days* and *cancer* for the evaluation of the publication bias.

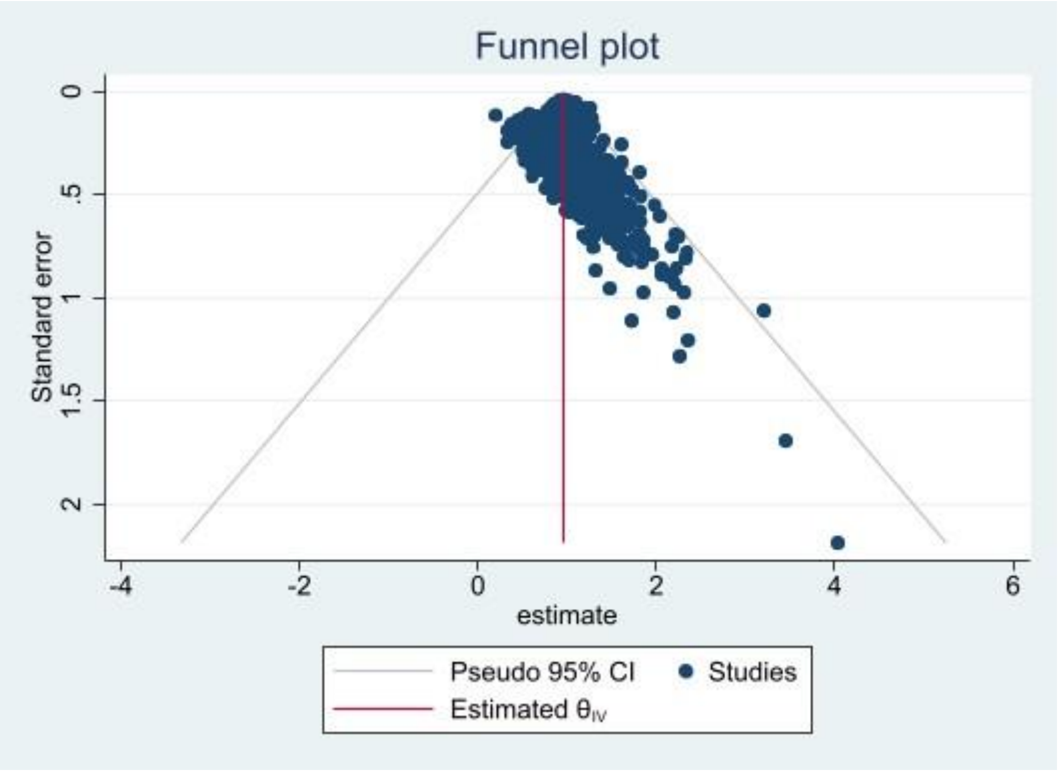

Figure S3. Funnel plot on association between *Intensity-weighted exposure days* and *cancer* for the evaluation of the publication bias.

Table S1. 71 Articles included in the meta-analysis with additional data extracted.

Authors	Title	Publication year	Study design	Health outcome type	Health outcome assessment type	Study location (country)	Crop type	Study period	Study population (N)	Active substances
Alavanja et al. [69]	Use of agricultural pesticides and prostate cancer risk in the Agricultural Health Study cohort	2003	Cohort (AHS)	Cancer	Population-based cancer registries	USA	ND	1993-1999	55,332 male pesticide applicators (private and commercial) (556 incident prostate cancers)	50 active ingredients analyzed: <u>Herbicides:</u> Alachlor, Atrazine, Butylate, Chlorimuron-ethyl, Cyanazine, Dicamba, 2,4-D, EPTC, Glyphosate, Imazethypyr, Metolachlor, Metribuzin, Paraquat, Pendimethalin, Petroleum oil as herbicide, 2,4,5-T, 2,4,5-TP, Trifluralin <u>Insecticides:</u> Aldicarb, Aldrin, Carbofuran, Carbaryl, Chlordane, Chlorpyrifos, Coumaphos, Dichlorvos, Diazinon, Dieldrin, DDT, Fonofos, Heptachlor, Lindane, Malathion, Parathion, Permethrin (for crops), Permethrin (for animals), Phorate, Terbufos, Toxaphene, Trichlorofon <u>Fungicides:</u> Benomyl, Captan, Chlorothalonil, Maneb/macozeb, Metalaxyl, Ziram <u>Fumigants:</u> Aluminum phosphide, Ethylene dibromide, Carbon tetrachloride/carbon disulfide, Methyl bromide
Alavanja et al. [70]	Pesticides and lung cancer risk in the Agricultural Health Study cohort	2004	Cohort (AHS)	Cancer	Population-based cancer registries	USA	ND	1993-2001	57,284 pesticide applicators and 32,333 spouses of farmer applicators (240 lung	<u>Herbicides:</u> Benzoic acid (Dicamba), Chloroacetanilide (Metolachlor), Dinitroaniline (Pendimethalin) <u>Insecticides:</u> Carbamate (Carbofuran), Phosphorothioate (Chlorpyrifos, Diazinon), Chlorinated organic (Dieldrin)

									cancer cases in apllicators and 60 in spouses)	
Alavanja et al. [71]	Non-Hodgkin Lymphoma risk and insecticide, fungicide and fumigant use in the Agricultural Health Study	2014	Cohort (AHS)	Cancer	Cancer registry files in Iowa and North Carolina	USA	ND	1993-2011	54,306 pesticide applicators (523 incident Non-hodgkin lymphomas)	26 active substances:<br><u>Insecticides:</u> Carbamate (Aldicarb, Carbaryl, Carbofuran), Organochlorine (Aldrin, Chlordane, DDT, Dieldrin, Heptachlor, Lindane, Toxaphene), Oganophosphate (Chlorpyrifos, Coumaphos, Dichlorvos, Diazinon, Fonofos, Malathion, Parathion, Phorate, Terbufos), Pyrethroid (Permethrin crops and animals)<br><u>Fungicides:</u> Acylalanine (Metalaxyl), Carbamate (Benomyl), Dithiocarbamate (Maneb/macozeb), Phthalimide (Captan), Polychlorinated aromatic thalonitrile (Chlorothalonil)<br><u>Fumigants:</u> Methyl halide (Methyl bromide)
Andreotti et al. [72]	Agricultural pesticide use and pancreatic cancer risk in the Agricultural Health Study cohort	2009	Cohort (AHS)	Cancer	Population-based cancer registries of Iowa and North Carolina	USA	ND	1993-2004	82,503 participants (52,721 pesticide applicators and 29,782 spouses) (93 incident cancer cases)	48 active ingredients:<br><u>Herbicides:</u> Benzoic (Dicamba), Chloroacetanilide (Alachlor, Metolachlor), Dinitroaniline (Pendimethalin, Trifluralin), Imidazolinone (Imazethypyr), Phenoxy (2,4-D, 2,4,5-T, 2,4,5-TP), Phosphinic (Glyphosate), Thiocarbamate (Butylate, EPTC), Triazine (Atrazine, Cyanazine, Metribuzin), Urea (Chlorimuron-ethyl), other herbicides (Paraquat, Petroleum oil)<br><u>Insecticides:</u> Carbamate (Carbaryl, Carbofuran), Organochlorine (Aldrin, Chlordane, DDT, Lindane, Heptachlor, Toxaphene), Oganophosphate (Chlorpyrifos, Coumaphos, Dichlorvos, Diazinon, Fonofos,

										Malathion, Parathion, Phorate, Terbufos, Trichlorofon), Pyrethroid (Permethrin crops and animals)<br>Fungicides: Benomyl, Captan, Chlorothalonil, Maneb/macozeb, Metalaxyl, Ziram<br>Fumigants: Aluminum phosphide, Ethylene dibromide, Carbon tetrachloride, Methyl bromide
Andreotti et al. [73]	Glyphosate use and cancer incidence in the Agricultural Health Study	2018	Cohort (AHS)	Cancer	Cancer registry files in Iowa and North Carolina	USA	ND	1993-2013	54,251 pesticide applicators (44,932 reported using Glyphosate) (5,779 incident cancers)	Glyphosate and 2,4-D, Alachlor, Atrazine, Metolachlor, Trifluralin, Lindane, DDT, Diazinon, Terbufos, Permethrin
Andreotti et al. [16]	Occupational pesticide use and risk of renal cell carcinoma in the Agricultural Health Study	2020	Cohort (AHS)	Cancer	Cancer registry files in Iowa and North Carolina	USA	ND	1993-2015	55,873 pesticide applicators (308 incident renal cell carcinomas)	37 active substances:<br>Herbicides: Anilides/anilines (Alachlor, Metolachlor, Pendimethalin, Trifluralin), Benzoic (Dicamba), Imidazolinone (Imazethypyr), Phenoxy (2,4-D, 2,4,5-T), Phosphinic (Glyphosate), Thiocarbamate (Butylate, EPTC), Triazine/triazinone (Atrazine, Cyanazine, Metribuzin), Urea (Chlorimuron ethyl), others (Paraquat, Petroleum oil)<br>Insecticides: Carbamate (Carbaryl, Carbofuran), Organochlorines (Aldrin, Chlordane, DDT, Heptachlor, Lindane, Toxaphene), Oganophosphate (Chlorpyrifos, Diazinon, Dichlorvos, Fonofos, Malathion, Phorate, Terbufos), Pyrethroid (Permethrin crops and animals)<br>Fungicides: Captan, Chlorothalonil, Metalaxyl<br>Fumigant: Methyl bromide

Band et al. [74]	Prostate cancer risk and exposure to pesticides in British Columbia farmers	2011	Case-control study	Cancer	Britisch Columbia Cancer Reegistry	Canada	45 animal and crop commodities	1983-1990	1,516 prostate cancers (113 farmers) and 4,994 controls (316 farmers) (all other cancer sites except lung and unknown primary site)	Assessment made for 290 different chemical agents, including 180 pesticides (see Table II for the list of active ingredients): 68 insecticides, 39 fungicides, 53 herbicides 50 others (fumigants, rodenticides, and plant growth regulators)
Barry et al. [75]	Methyl bromide exposure and cancer risk in the Agricultural Health Study	2012	Cohort (AHS)	Cancer	Cancer registry files in Iowa and North Carolina	USA	ND	1993-2007	53,588 pesticide applicators (7,814 reported using Methyl bromide) (2,960 incident cancer cases)	Methyl bromide and Metalaxyl, Ethylene dibromide, Carbaryl, Aldicarb, Maneb/mancozeb
Beane Freeman et al. [76]	Cancer incidence among male pesticide applicators in the Agricultural Health Study cohort exposed to Diazinon	2005	Cohort (AHS)	Cancer	Cancer registry files in Iowa and North Carolina	USA	ND	1993-2002	23,106 male applicators (301 incident cancer cases)	Diazinon
Bonner et al. [77]	Malathion exposure and the incidence of caancer in the Agricultural Health Study	2007	Cohort (AHS)	Cancer	Population-based tumor registries of Iowa and North Carolina	USA	Only analysis of Alfalfa and cotton productions	1993-2002	19,717 pesticides applicators using malthion	Malathion and Carbaryl, Parathion, Diazinon, Chlordane, Lindane

Bonner et al. [78]	Occupational exposure to Terbufos and the incidence of cancer in the Agricultural Health Study	2010	Cohort (AHS)	Cancer	Cancer registry files in Iowa and North Carolina	USA	Only analysis of Corn production	1993-2005	57,310 pesticide applicators (16,489 reported using Terbufos) (2,960 incident cancer cases)	Terbufos and Carbofuran, Fonofos, Atrazine, 2,4-D, Phorate
Bonner et al. [79]	Occupational exposure to pesticides and the incidence of lung cancer in the Agricultural Health Study	2017	Cohort (AHS)	Cancer	Cancer registry files in Iowa and North Carolina	USA	ND	1993-2011	57,310 pesticide applicators (654 incident lung cancers)	Herbicides: Benzoic (Dicamba),Dinitroaniline (Pendimethalin), Pyrimidylsulfonylurea (Chlorimuron-ethyl) Insecticides: Carbamate (Carbaryl, Carbofuran), Organochlorine (Chlordane, DDT, Dieldrin, Heptachlor), Oganophosphate (Chlorpyrifos, Malathion, Parathion) Fungicdes: Dithiocarbamate (Maneb/macozeb)
Boulanger et al. [62]	Agricultural exposure and risk of bladder cancer in the AGRIculture and CANcer cohort	2017	Cohort (AGRICAN)	Cancer	Cancer registries	France	13 types of crops + 5 types of livestock	2005-2009	148,051 women and men farm owners and workers (179 incident bladder cancers)	Arsenic
Boulanger et al. [80]	Lung cancer risk and occupational exposures in crop farming: results from the AGRIculture and CANcer (AGRICAN) cohort	2018	Cohort (AGRICAN)	Cancer	Cancer registries	France	13 types of crops + 5 types of livestock	2005-2013	148,044 women and men active and retired in agriculture-related activities (897 incident	ND

									cases of lung cancers)	
Chiu et al. [81]	Agricultural pesticide use and risk of t(14;18)-defined subtypes of non-Hodgkin lymphoma	2006	Case-control study	Cancer	Pathology review and histology for cases from the Nebraska Lymphoma Study Group and hospitals	USA	ND	1983-1986	172 non-Hodgkin lymphoma cases (65 t(14;18)-positive and 107 t(14;18)-negative) and 1,432 controls (no hematopoietic cancers)	<u>Insecticides:</u> Organochlorines, Carbamates, Organophosphates, Pyrethropids (only family groups no details on active substances) <u>Herbicides:</u> Phenoxyacetic acids, Triazines, Amides, Benzoic acids, Carbamates, Dinitroanilines (only family groups no details on active substances)
Christensen et al. [82]	Coumaphos exposure and incident cancer among male participants in the Agricultural Health Study (AHS)	2010	Cohort (AHS)	Cancer	Cancer registry files in Iowa and North Carolina	USA	Only analysis of Cattle farm	1993-2005	47,822 male pesticide applicators (3,689 reported using Coumaphos) (2,960 incident cancer cases)	Coumaphos
De Roos et al. [83]	Cancer incidence among Glyphosate-exposed pesticide applicators in the Agricultural Health Study	2005	Cohort (AHS)	Cancer	Cancer registry files in Iowa and North Carolina	USA	ND	1993-2001	52,656 pesticide applicators (private and commercial) (52,207 males and 1,449 females)	Glyphosate and 2,4-D, Alachlor, Atrazine, Metolachlor, Trifluralin, Carbaryl, Benomyl, Maneb, Paraquat, Diazinon
Delancey et al. [84]	Occupational exposure to Metribuzin and the incidence of cancer in the	2009	Cohort (AHS)	Cancer	Population-based cancer registries of Iowa and	USA	ND	1993-2004	23,072 pesticide applicators (8,504	Metribuzin and Cyanazine, Chlorimuron ethyl, Pendimethalin, Butylate, Trifluralin

	Agricultural Health Study				North Carolina				reported using Metribuzin) (554 incident cancer cases)	
Dennis et al. [85]	Pesticide use and cutaneous melanoma in pesticide applicators in the Agricultural Heath Study	2010	Cohort (AHS)	Cancer	Cancer registry files in Iowa and North Carolina	USA	ND	1993-2005	24,704 pesticide applicators (150 incident melanomas)	50 active ingredients: Herbicides: Alachlor, Atrazine, Butylate, Chlorimuron-ethyl, Cyanazine, Dicamba, 2,4-D, EPTC, Glyphosate, Imazethypyr, Metolachlor, Metribuzin, Paraquat, Pendimethalin, Petroleum oil, 2,4,5-T, 2,4,5-TP, Trifluralin Insecticides: Aldicarb, Aldrin, Carbaryl, Carbofuran, Chlordane, Chlorpyrifos, Coumaphos, DDT, Diazinon, Dichlorvos, Dieldrin, Fonofos, Heptachlor, Lindane, Malathion, Parathion, Permethrin (for crops), Permethrin (for animals), Phorate, Terbufos, Toxaphene, Trichlorofon Fungicides: Benomyl, Captan, Chlorothalonil, Maneb/macozeb, Metalaxyl, Ziram Fumigants: Aluminum phosphide, Ethylene dibromide, Carbon tetrachloride/carbon disulfide, Methyl bromide
Engel et al. [86]	Pesticide use and breast cancer risk among farmers' wives in the Agricultural Health Study	2005	Cohort (AHS)	Cancer	Population-based cancer registries	USA	ND	1993-2000	30,454 women (wives of private pesticide applicators) (309 incident breast cancer cases)	47 active ingredients: Herbicides: Alachlor, Atrazine, Butylate, Chlorimuron-ethyl, Cyanazine, Dicamba, EPTC, Glyphosate, Imazethypyr, Metolachlor, Metribuzin, Paraquat, Pendimethalin, Petroleum oil, Trifluralin, 2,4-D, 2,4,5-T, 2,4,5-TP Insecticides: Aldicarb, Aldrin, Carbaryl, Carbofuran, Chlordane, Chlorpyrifos, Coumaphos, Dichlorvos, Diazinon, Dieldrin, DDT, Fonofos, Heptachlor, Lindane,

										Malathion, Parathion, Permethrin (for crops), Permethrin (for animals), Phorate, Terbufos, Toxaphene<br>Fungicides: Benomyl, Captan,Chlorothalonil, Maneb, Metalaxyl<br>Fumigants: Ethylene dibromide, Carbon tetrachloride/carbon disulfide, Methyl bromide
Goldner et al. [87]	Hypothyroidism and pesticide use among male private pesticide applicators in the agricultural health study	2013	Cohort (AHS)	Endocrine disruption (Thyroid)	Self-reported questionnaire and telephone interviews for follow-up	USA	ND	1993-2010	22,246 male applicators	50 active ingredients:<br>Herbicides: Benzoic (Dicamba), Chloroacetanilide (Alachlor, Metolachlor), Dinitroaniline (Pendimethalin, Trifluralin), Imidazolinone (Imazethypyr), Phenoxy (2,4-D, 2,4,5-T, 2,4,5-TP), Phosphinic (Glyphosate), Thiocarbamate (Butylate, EPTC), Triazine (Atrazine, Cyanazine, Metribuzin), Urea (Chlorimuron-ethyl), other herbicides (Paraquat, Petroleum oil)<br>Insecticides: Carbamate (Aldicarb, Carbaryl, Carbofuran), Organochlorine (Aldrin, Chlordane, Dieldrin, DDT, Lindane, Heptachlor, Toxaphene), Oganophosphate (Chlorpyrifos, Coumaphos, Diazinon, Dichlorvos, Fonofos, Malathion, Parathion, Phorate, Terbufos, Trichlorphon), Pyrethroid (Permethrin crops and animals)<br>Fungicides: Benomyl, Captan, Chlorothalonil, Maneb/Mancozeb, Metalaxyl, Ziram<br>Fumigants: Aluminum phosphide, Ethylene dibromide, Carbon disulfide/Carbon tetrachloride, Methyl bromide
Greenburg et al. [88]	Cancer incidence among pesticide applicators exposed to Captan in	2008	Cohort (AHS)	Cancer	Cancer registry files in Iowa and	USA	Only analysis of Field corn and	1993-2004	48,986 pesticide applicators (4,383	Captan

	the Agricultural Health Study				North Carolina		Soybean productions		reported using Captan)	
Hofmann et al. [89]	Lifetime pesticide use and monoclonal gammopathy of undetermined significance in a prospective cohort of male farmers	2021	Cohort (AHS)	Cancer	Biomonitoring (MGUS assessment)	USA	ND	1993-2010	1,638 male farmers	36 pesticides (16 insecticides, 17 herbicides, 2 fungicides, 1 fumigant): <u>Insecticides:</u> Organochlorine insecticides (Aldrin, Aldrin/dieldrin combined, Chlordane, DDT, Heptachlor, Lindane), Organophosphate insecticides (Chlorpyrifos, Coumaphos, Diazinon, Dichlorvos, Fonofos, Malathion, Phorate, Terbufos), Carbamate insecticides (Carbaryl, Carbofuran), Pyrethroid insecticides (Permethrin) <u>Herbicides:</u> 2,4-D, 2,4,5-T, Alachlor, Atrazine, Butylate, Chlorimuron ethyl, Cyanazine, Dicamba, EPTC, Glyphosate, Imazethapyr, Metolachlor, Metribuzin, Paraquat, Pendimethalin, Petroleum oil/distillates, Trifluralin <u>Fungicides:</u> Captan, Metalaxyl <u>Fumigants:</u> Methyl bromide
Hou et al. [90]	Pendimethalin exposure and cancer incidence among pesticide applicators	2006	Cohort (AHS)	Cancer	Cancer registry files in Iowa and North Carolina	USA	ND	1993-2002	57,311 pesticide applicators and 32,347 spouses of private applicators (9,089 pendimethalin-exposed and 15,285 non-pendimethalin-exposed applicators)	Pendimethalin and Ziram, Dieldrin, Butylate, Chlorimuron-ethyl, Metribuzin

Ji et al. [91]	Occupational exposure to pesticides and pancreatic cancer	2001	Case-control study	Cancer	Cancer registries (cases diagnosed by tissue confirmation)	USA	ND	1986-1989	485 pancreatic cancer cases and 2,109 controls (general population)	Only the three main categories (i.e., insecticides, fungicides, and herbicides), without details on active ingredients, were used in exposure assessment
Jones et al. [92]	Incidence of solid tumours among pesticide applicators exposed to the organophosphate insecticide diazinon in the Agricultural Health Study: an updated analysis	2015	Cohort (AHS)	Cancer	Cancer registry files in Iowa and North Carolina	USA	Only analysis of livestock and poultry farms	1993-2011	22,830 male pesticide applicators (5,120 reported using Methyl bromide) (2,288 incident solid tumours)	Diazinon and Chlorpyrifos, Dicamba, Dieldrin, Metolachlor, Pendimethalin, Carbofuran, Terbufos
Kamel et al. [93]	Neurologic symptoms in licensed pesticide applicators in the Agricultural Health Study	2007	Cohort (AHS)	Neurotoxicity	Self-reported questionnaire	USA	ND	1993-1997	18,782 male applicators	Insecticides (Organophosphates, organochlorines, carbamates, pyrethroids) Fumigants
Kang et al. [94]	Cancer incidence among pesticide applicators exposed to Trifluralin in the Agricultural Health Study	2008	Cohort (AHS)	Cancer	Cancer registry files in Iowa and North Carolina	USA	ND	1993-2002	50,127 pesticide applicators (25,712 reported using Trifluralin)	Trifluralin and Dicamba, Metolachlor, Imazethayr, Metribuzin, Cyanazine
Koutros et al. [95]	Dichlorvos exposure and human cancer risk: Results from the Agricultural Health Study	2008	Cohort (AHS)	Cancer	Cancer registry files in Iowa and North Carolina	USA	ND	1993-2004	57,311 pesticide applicators (4,613 reported using DDVP)	Dichlorvos (DDVP)
Koutros et al. [96]	Aromatic amine pesticide use and	2009	Cohort (AHS)	Cancer	Cancer registry files in	USA	ND	1993-2004	49,398 pesticide	Imazethapyr and Permethrin (crops), Fonofos, Trichlorfon, Carbofuran, Glyphosate,

	human cancer risk: results from the U.S. Agricultural Health Study				Iowa and North Carolina				applicators (20,646 reported using Imazethapyr) (2,907 incident cancer cases)	Metolachlor, EPTC, Alachlor, Pendimethalin, and Trifluralin
Koutros et al. [97]	Risk of total and aggressive Prostate Cancer and pesticide use in the Agricultural Health Study	2013	Cohort (AHS)	Cancer	Cancer registry files in Iowa and North Carolina	USA	ND	1993-2007	54,412 pesticide applicators (1,962 incident prostate cancers)	18 active substances: <u>Insecticides:</u> Organochlorine (Aldrin, Chlordane, DDT, Dieldrin, Lindane, Heptachlor, Toxaphene), Oganophosphate (Chlorpyrifos, Coumaphos, Dichlorvos, Diazinon, Fonofos, Malathion, Parathion, Phorate, Terbufos) <u>Herbicides:</u> Triazine (Atrazine, Cyanazine)
Koutros et al. [98]	Occupational exposure to pesticides and bladder cancer risk	2016	Cohort (AHS)	Cancer	Cancer registry files in Iowa and North Carolina	USA	ND	1993-2011	57,310 pesticide applicators (321 incident bladder cancers)	36 active ingredients: <u>Herbicides:</u> 2,4,5-T, 2,4-D, Alachlor, Atrazine, Butylate, Chlorimuron-ethyl, Cyanazine, Dicamba, EPTC, Glyphosate, Imazethypyr, Metolachlor, Metribuzin, Paraquat, Pendimethalin, Petroleum oil, Trifluralin <u>Insecticides:</u> Aldicarb, Aldrin, Carbaryl, Carbofuran, Chlordane, Chlorpyrifos, Coumaphos, Diazinon, DDT, Dichlorvos, Fonofos, Heptachlor, Lindane, Malathion, Parathion, Permethrin, Phorate, Terbufos, Toxaphene
Lee et al. [99]	Cancer incidence among pesticide applicators exposed to alachlor in the Agricultural Health Study	2004	Cohort (AHS)	Cancer	Cancer registry files in Iowa and North Carolina	USA	Only analysis of Corn production	1993-2000	49,980 pesticide applicators (1,466 incident malignant neoplasms)	Alachlor and Atrazine, Cyanazine, Metolachlor, Trifluralin, 2,4-D

Lee et al. [100]	Cancer incidence among pesticide applicators exposed to Chlorpyrifos in the Agricultural Health Study	2004	Cohort (AHS)	Cancer	Cancer registry files in Iowa and North Carolina	USA	ND	1993-2001	54,383 pesticide applicators (52,910 males and 1,473 females) (2,070 incident malignant neoplasms)	Chlorpyrifos and Alachlor, Carbofuran, Fonofos, Trifluralin
Lee et al. [101]	Pesticide use and colorectal cancer risk in the Agricultural Health Study	2007	Cohort (AHS)	Cancer	Population-based cancer registries of Iowa and North Carolina	USA	ND	1993-2002	56,813 pesticide applicators (305 incident colorectal cancers)	50 active ingredients: <u>Herbicides:</u> Alachlor, Atrazine, Butylate, Chlorimuron-ethyl, Cyanazine, Dicamba, 2,4-D, EPTC, Glyphosate, Imazethypyr, Metolachlor, Metribuzin, Paraquat, Pendimethalin, Petroleum oil/distillate, 2,4,5-T, 2,4,5-TP, Trifluralin <u>Insecticides:</u> Aldicarb, Aldrin, Carbaryl, Carbofuran, Chlordane, Chlorpyrifos, Coumaphos, DDT, Diazinon, Dichlorvos/DDVP, Dieldrin, Fonofos, Heptachlor, Lindane, Malathion, Parathion, Permethrin (for crops), Permethrin (for animals), Phorate, Terbufos, Toxaphene, Trichlorofon <u>Fungicides:</u> Benomyl, Captan, Chlorothalonil, Maneb/macozeb, Metalaxyl, Ziram <u>Fumigants:</u> Aluminum phosphide, Ethylene dibromide, Carbon tetrachloride/carbon disulfide, Methyl bromide
Lemarchand et al. [102]	Prostate cancer risk among French farmers in the AGRICAN cohort	2016	Cohort (AGRICAN)	Cancer	Cancer registries	France	13 types of crops (Wheat and/or Barley, Vineyard, Corn,	2005-2009	81,961 men active and retired in agriculture-related activities	ND

							Potato, Beet, Fruits, Tobacco, Field-grown vegetable, Rape, Sunflower, Peas, Grenn-house) + 5 types of livestock (Cattle, Poultry, Pigs, Horses, Sheep and/or goats)		(1496 incident prostate cancers)	
Lerro et al. [103]	Use of Acetochlor and cancer incidence in the Agricultural Health Study	2015	Cohort (AHS)	Cancer	Cancer registry files in Iowa and North Carolina	USA	ND	1993-2011	33,484 male pesticide applicators (4,026 reported using Acetochlor) (3,234 incident cancers)	Acetochlor and Imazethapyr, Dicamba, Atrazine, EPTC, Cyanazine, Pendimethalin, Trifluralin, Flumetsulam, Clopyralid, Isoxaflutole, Alachlor, Metolachlor, Atrazine, EPTC, 2,4-D, Butylate, Aldicarb, Chlordane, Chlorpyrifos, Toxaphene
Lerro et al. [104]	Dicamba use and cancer incidence in the Agricultural Health Study: an updated analysis	2020	Cohort (AHS)	Cancer	Cancer registry files in Iowa and North Carolina	USA	Only analysis of Beef cattle, hog, and poultry farms, field corn, cotton, wheat, and	1993-2015	49,922 pesticide applicators (26,412 reported using Dicamba) (5,779 incident cancers)	Dicamba and Imazethapyr, 2,4-D, Trifluralin, Atrazine and Cyanazine.

							soybean productions			
Lerro et al. [105]	Pesticide exposure and incident thyroid cancer among male pesticide applicators in Agricultural Health Study	2021	Cohort (AHS)	Cancer	Cancer registry files in Iowa and North Carolina	USA	Only analysis of Cattle and hog farms, Alfalfa, Field corn, Soybean and Tobacco productions	1993-2015	53,096 male pesticide applicators (85 incident thyroid cancers)	44 active substances: <u>Herbicides:</u> Chloroacetanilides (Alachlor, Metolachlor), Dinitroaniline (Pendimethalin, Trifluralin), Benzoic (Dicamba), Imidazolinone (Imazethypyr), Phenoxy (2,4-D, 2,4,5-T, 2,4,5-TP), Phosphinic (Glyphosate), Thiocarbamate (Butylate, EPTC), Triazine/triazinone (Atrazine, Cyanazine, Metribuzin), Urea (Chlorimuron ethyl), others (Paraquat, Petroleum oil) <u>Insecticides:</u> Carbamate (Aldicarb, Carbaryl, Carbofuran), Organochlorines (Aldrin, Chlordane, DDT, Heptachlor, Lindane, Toxaphene), Oganophosphate (Chlorpyrifos, Coumaphos, Diazinon, Dichlorvos, Fonofos, Malathion, Parathion, Phorate, Terbufos), Pyrethroid (Permethrin) <u>Fungicides:</u> Benomyl, Captan, Chlorothalonil, Maneb/Mancozeb, Metalaxyl <u>Fumigant:</u> Aluminum Phosphide, Methyl bromide
Lerro et al. [106]	Occupational pesticide exposure and subclinical hypothyroidism among male pesticide applicators	2018	Cohort (AHS)	Endocrine disruption (Thyroid)	Self-reported questionnaire and blood sampling	USA	ND	2010-2013	619 male applicators	30 active ingredients: <u>Herbicides:</u> Benzoic (Dicamba), Chloroacetanilide (Alachlor, Metolachlor), Dinitroaniline (Pendimethalin, Trifluralin), Imidazolinone (Imazethypyr), Phenoxy (2,4-D, 2,4,5-T), Phosphinic (Glyphosate), Thiocarbamate (Butylate, EPTC), Triazine (Atrazine, Cyanazine, Metribuzin), Urea (Chlorimuron-ethyl), other herbicides (Petroleum distillates) <u>Insecticides:</u> Carbamate (Carbaryl,

										Carbofuran), Organochlorine (Aldrin, Chlordane, DDT, Heptachlor), Oganophosphate (Chlorpyrifos, Diazinon, Fonofos, Malathion, Phorate, Terbufos), Pyrethroid (Permethrin) Fungicides: Captan, Metalaxyl Fumigants: Carbon disulfide/Carbon tetrachloride, Methyl bromide
Lynch et al. [107]	Cancer incidence among pesticide applicators exposed to Cyanazine in the Agricultural Health Study	2006	Cohort (AHS)	Cancer	Cancer registry files in Iowa and North Carolina	USA	Only analysis of Corn production	1993-2002	57,311 pesticide applicators (20,341 reported ever using Cyanazine)	Cyanazine and Metolachlor, EPTC, Alachlor, Imazethapyr, Trifluralin
Lynch et al. [108]	Cancer incidence among pesticide applicators exposed to Butylate in the agricultural health study (AHS)	2009	Cohort (AHS)	Cancer	Cancer registry files in Iowa and North Carolina	USA	ND	1993-2004	19,655 pesticide applicators (5,297 reported using Butylate) (2,907 incident cancer cases)	Butylate and Atrazine, Metribuzin, Chlorimuron-ethyl, Pendimethalin, Aldrin, Ziram
Mahajan et al. [109]	Phorate exposure and incidence of cancer in the Agricultural Health Study	2006	Cohort (AHS)	Cancer	Population-based tumor registries of Iowa and North Carolina	USA	Only analysis of Corn production	1993-2002	52,395 private applicators (farmers) and 4,916 commercial applicators	Phorate and Aldicarb, Ethylene dibromide, Aldrin, 2,4,5-TP, Butylate
Mahajan et al. [110]	Fonofos exposure and cancer incidence in the Agricultural Health Study	2006	Cohort (AHS)	Cancer	Population-based tumor registries of Iowa and North Carolina	USA	Only analysis of Corn production and of the number of livestock on	1993-2002	45,372 pesticide applicators	Fonofos and Trichorfon, Carbofuran, Imazethapyr, EPTC

							farm (other than poultry)		
Mahajan et al. [111]	Carbaryl exposure and incident cancer in the Agricultural Health Study	2007	Cohort (AHS)	Cancer	Population-based tumor registries of Iowa and North Carolina	USA	ND	1993-2002	21,416 pesticides applicators (1,291 incident cancer cases)	Carbaryl and Diazinon, Chlordane, Malathion, Dieldrin
Mills and Yang [112]	Prostate cancer risk in California farm workers	2003	Case-control study from Cohort (UFW)	Cancer	California Cancer Registry	USA	Grapes/Tree Fruit, Citrus fruit, Vegetables, Mushrooms, Strawberries, Horticulture	1988-1999	222 prostate cancers in Hispanic male farm workers and 1,110 controls (cancer-free Hispanic male farm workers)	Chlorothalonil, Diazinon, Dichloropropene, Dichlorvos, Dicofol, Heptachlor, Lindane, Malathion, Mancozeb, Maneb, Methyl bromide, Propargite, Propoxur, Propyzamide, Simazine, Trifluralin
Mills et al. [113]	Lymphohematopoietic cancers in the United Farm Workers of America (UFW), 1988–2001	2005	Case-control study from Cohort (UFW)	Cancer	California Cancer Registry	USA	Grapes, Citrus, Vegetables, Mushrooms, Strawberries, Horticulture	1987-2001	139,000 farm workers (131 Lymphohematopoietic cancers in Hispanic workers (94 males; 37 females) and 655 controls in Hispanic farmers)	2,4-D, Captan, Chlorothalonil, Diazinon, Dichloropropene, Malathion, Mancozeb, Maneb, Methyl bromide, Methyl parathion, Nitrofen, Propyzamide, Simazine, Toxaphene, Trifluralin
Montgomery et al. [114]	Incident Diabetes and Pesticide Exposure among Licensed Pesticide	2008	Cohort (AHS)	Endocrine disruption (Diabete)	Self-reported questionnaire and telephone	USA	ND	1993-2003	31,787 applicators (30,955 males and 832 females)	50 active ingredients: <u>Herbicides:</u> Benzoic (Dicamba), Chloroacetanilide (Alachlor, Metolachlor), Dinitroaniline (Pendimethalin, Trifluralin),

	Applicators: Agricultural Health Study 1993 – 2003				interviews for follow-up					Imidazolinone (Imazethypyr), Phenoxy (2,4-D, 2,4,5-T, 2,4,5-TP), Phosphinic (Glyphosate), Thiocarbamate (Butylate, EPTC), Triazine (Atrazine, Cyanazine, Metribuzin), Urea (Chlorimuron-ethyl), other herbicides (Paraquat, Petroleum oil) <u>Insecticides:</u> Carbamate (Aldicarb, Carbaryl, Carbofuran), Organochlorine (Aldrin, Chlordane, DDT, Dieldrin, Heptachlor, Lindane, Toxaphene), Oganophosphate (Chlorpyrifos, Coumaphos, Diazinon, Dichlorvos, Fonofos, Malathion, Parathion, Phorate, Terbufos, Trichlorphon), Pyrethroid (Permethrin crops and animals) <u>Fungicides:</u> Benomyl, Captan, Chlorothalonil, Maneb, Metalaxyl, Ziram <u>Fumigants:</u> Aluminum phosphide, Ethylene dibromide,Carbon tetrachloride, Methyl bromide
Mozzachio et al. [115]	Chlorothalonil exposure and cancer incidence among pesticide applicator participants in the Agricultural Health Study	2008	Cohort (AHS)	Cancer	Cancer registry files in Iowa and North Carolina	USA	ND	1993-2004	47,625 pesticide applicators (3,657 reported using Chlorothalonil)	Chlorothalonil and Dieldrin, Heptachlor, Ziram
Narayan et al. [116]	Occupational pesticide use and Parkinson's disease in the Parkinson Environment Gene (PEG) Study	2017	Case-control study	Neurotoxicity	Diagnosed	USA	ND	2001-2011	360 cases (PD) and 827 controls	ND
Pardo et al. [117]	Pesticide exposure and risk of aggressive prostate cancer	2020	Cohort (AHS)	Cancer	Cancer registry files in Iowa and	USA	ND	1993-2015	20,923 male pesticide private	38 active substances: <u>Herbicides:</u> Amide (Acetochlor, Dimethenamid), Amide/Diphenyl ether

	among private pesticide applicators				North Carolina				applicators (883 incident aggressive prostate cancers)	(Fomesafen), Amide/Triazolopyrimidine (Cloransulam-methyl, Flumetsulam), Benzoylcyclohexanedione (Mesotrione), Cyclohexene oxime (Clethodim, Sethoxydim), Diphenyl ether (Acifluorfen), Growth inhibitor/Gametocides (Maleic hydrazide), Imidazolinone (Imazaquin), Nitrile (Bromoxynil), Organophosphorous (Glufosinate-ammonium), Oxazole (Clomazone), Oxazole/Cyclopropylisoxazole (Isoxaflutole), Phenoxy (Fenoxaprop-p-ethyl, Fluazifop-butyl), Pyridine/Aromatic acid (Clopyralid, Picloram), Pyridine (Triclopyr), Urea (Linuron, Nicosulfuron, Rimsulfuron, Thifensulfuron-methyl), other (Sodium bentazon) <u>Insecticides:</u> Carbamate (Methomyl), Organochlorine(Endosulfan), Organophosphorous (Acephate, Dimethoate, Disulfoton, Tebupirimfos), Pyrethroid (Cyfluthrin, Lambda-cyhalothrin, Tefluthrin), Others (Chloropicrin, Bacillus thuringiensis) <u>Nematicides:</u> Fumigant (1, 3-dichloropropene) <u>Other:</u> Arsenical pesticides (lead arsenate (insecticide), organic arsenic (herbicide), and inorganic arsenic (herbicide))
Park et al. [118]	Cancer incidence among paraquat-exposed pesticide applicators in the Agricultural Health Study	2009	Cohort (AHS)	Cancer	Cancer registry files in Iowa and North Carolina	USA	Only analysis of Corn production	1993-2004	56,222 pesticide applicators (11,256 reported using Paraquat)	Paraquat and Metribuzin, Pendimenthalin, 2,4,5-TP, Butylate, Dieldrin

Park et al. [119]	Exposure to pesticides and the prevalence of diabetes in a rural population in Korea	2019	Korea Farmers Cohort	Endocrine disruption (Diabete)	Questionnaires in a face-to-face interview, clinical examination, and blood sampling	Korea	ND	2005-2008	2,559 farmers and farm managers (1,048 males and 1,511 females)	ND
Piel et al. [64]	Central nervous system tumors and agricultural exposures in the prospective cohort AGRICAN	2017	Cohort (AGRI-CAN)	Cancer	Cancer registries	France	13 types of crops + 5 types of livestock	2005-2011	170,858 women and men active and retired in agriculture-related activities (273 incident cases of CNS tumors)	ND
Piel et al. [120]	Increased risk of central nervous system tumours with carbamate insecticide use in the prospective cohort AGRICAN	2019	Cohort (AGRI-CAN)	Cancer	Cancer registries	France	13 types of crops + 5 types of livestock	2005-2013	170,858 women and men active and retired in agriculture-related activities (381 incident cases of CNS tumors)	19 carbamates insecticides (aldicarb, bendiocarb, benfuracarb, carbaryl, carbofuran, carbosulfan, dimetilan, dioxacarb, ethiofencarb, fenoxycarb, formetanate,furathiocarb, isolan, methiocarb, methomyl, pirimicarb, promecarb, thiodicarb and thiofanox)
Piel et al. [121]	Agricultural exposures to carbamate herbicides and fungicides and central nervous system tumour incidence in	2019	Cohort (AGRI-CAN)	Cancer	Cancer registries	France	13 types of crops + 5 types of livestock	2005-2013	170,858 women and men active and retired in agriculture-related activities	7 carbamate herbicides (asulam, barban, chlorbufam, chlorpropham, desmedipham, phenmedipham, propham)<br>7 thiocarbamate herbicides (butylate, cycloate, diallate, EPTC, prosulfocarb, triallate, vernolate)<br>3 carbamate fungicides (diethofencarb,

	the cohort AGRI-CAN								(381 incident cases of CNS tumors)	iprovalicarb, propamocarb) 11 dithiocarbamate fungicides (cupreb, cuprobam, ferbam, mancopper, mancozeb, maneb, metiram, propineb, thiram, zineb, ziram)
Pouchieu et al. [65]	Pesticide use in agriculture and Parkinson’s disease in the AGRICAN cohort study	2018	Cohort (AGRI-CAN)	Neurotoxicity	Self-reported Parkinson's disease (PD)	France	13 crops (grassland, wheat and/or barley, vineyards, corn, potatoes, fruit-growing, beets, rape, peas, sunflower, tobacco, vegetables, and greenhouses), 5 types of livestock (cattle , poultry, swine , horses, and sheep/goats)	2005-2007	149,810 men active and retired in agriculture-related activities (1,732 incident cases of PD)	Dithiocarbamate fungicides (cupreb, ferbam, cuprobam, mancopper, mancozeb, maneb, metiram, propineb, thiram, zineb and ziram) Bipyridyle herbicides (diquat and paraquat) Insecticide rotenone
Purdue et al. [122]	Occupational exposure to organochlorine insecticides and cancer incidence in the Agricultural Health Study	2007	Cohort (AHS)	Cancer	Cancer registry files in Iowa and North Carolina	USA	ND	1993-2002	51,011 pesticide applicators (24,384 reported ever using organochlorine insecticides)	7 organochlorine insecticides (Aldrin, Chlordane, DDT, Dieldrin, Heptachlor, Lindane, Toxaphene)

Renier et al. [123]	Agricultural exposure and risk of soft tissue sarcomas and gastrointestinal stromal sarcoma in the AGRIculture and CANcer (AGRICAN) cohort	2022	Cohort (AGRICAN)	Cancer	Cancer registries	France	14 types of crops + 5 types of livestock	2005-2015	148,036 women (n=65,328) and men (n=82,708) active and retired in agriculture-related activities (188 incident cases of sarcomma: 74 women and 114 men)	3 phenoxyherbicides (i.e., 2,4-D; 2,4,5-T and MCPA) and pentachlorophenol
Rusiecki et al. [124]	Cancer incidence among pesticide applicators exposed to Atrazine in the Agricultural Health Study	2004	Cohort (AHS)	Cancer	Cancer registry files in Iowa and North Carolina	USA	Only analysis of Corn production	1993-2001	53,943 pesticide applicators (52,740 males)	Atrazine and Dicamba, Cyanazine, Alachlor, 2,4-D, Chlorimuronethyl, Metribuzin, Butylate, Phorate, Heptachlor
Rusiecki et al. [125]	Cancer incidence among pesticide applicators exposed to metolachlor in the Agricultural Health Study	2006	Cohort (AHS)	Cancer	Cancer registry files in Iowa and North Carolina	USA	Only analysis of Corn, Sorghum, and Soybean productions	1993-2002	50,193 pesticide applicators (males and females)	Metolachlor and Cyanazine, EPTC, Alachlor, Imazethapyr, Trifluralin
Rusiecki et al. [126]	Cancer incidence among pesticide applicators exposed to Permethrin in the Agricultural Health Study	2009	Cohort (AHS)	Cancer	Cancer registry files in Iowa and North Carolina	USA	ND	1993-2004	49,093 pesticide applicators (11,623 reported using Permethrin) (2,907	Permethrin and Trichlorfon, Ziram, Coumaphos, Aldicarb, Chlorothalonil, Dichlorvos, Cyanazine, Metolachlor, Atrazine, Alachlor

									incident cancer cases)	
Samanic et al. [127]	Cancer incidence among pesticide applicators exposed to Dicamba in the Agricultural Health Study	2006	Cohort (AHS)	Cancer	Cancer registry files in Iowa and North Carolina	USA	Only analysis of Soybean and field Corn productions	1993-2002	41,969 applicators (22,036 reported ever using Dicamba)	Dicamba and 2,4-D, Atrazine, Glyphosate
Shrestha et al. [128]	Pesticide use and incident Parkinson's disease in a cohort of farmers and their spouses	2020	Cohort (AHS)	Neurotoxicity	Self-reported questionnaire	USA	ND	1993-2016	38,274 applictors and 27,836 spouses (373 applicators and 118 spouses reported new Parkinson's disease	48 active ingredients:<br>Herbicides: Benzoic (Dicamba), Chloroacetanilide (Alachlor, Metolachlor), Dinitroaniline (Pendimethalin, Trifluralin), Imidazolinone (Imazethypyr), Phenoxy (2,4-D, 2,4,5-T, 2,4,5-TP), Phosphinic (Glyphosate), Thiocarbamate (Butylate, EPTC), Triazine (Atrazine, Cyanazine, Metribuzin), Urea (Chlorimuron-ethyl), other herbicides (Paraquat, Petroleum oil)<br>Insecticides: Carbamate (Aldicarb, Carbaryl, Carbofuran), Organochlorine (Aldrin, Chlordane, Dieldrin, DDT, Lindane, Heptachlor, Toxaphene), Oganophosphate (Chlorpyrifos, Coumaphos, Diazinon, Dichlorvos, Fonofos, Malathion, Parathion, Phorate, Terbufos), Pyrethroid (Permethrin crops and animals)<br>Fungicides: Benomyl, Captan, Chlorothalonil, Maneb, Metalaxyl<br>Fumigants: Aluminum phosphide, Ethylene dibromide, Carbon disulfide/Carbon tetrachloride, Methyl bromide
Shrestha et al. [129]	Pesticide use and incident hypothyroidism in pesticide applicators in	2018	Cohort (AHS)	Endocrine disruption (Thyroid)	Self-reported questionnaire and telephone	USA	ND	1993-2016	34,879 applicators (34,125 males and 754 females)	48 active ingredients:<br>Herbicides: Benzoic (Dicamba), Chloroacetanilide (Alachlor, Metolachlor), Dinitroaniline (Pendimethalin, Trifluralin),

	the Agricultural Health Study				interviews for follow-up					Imidazolinone (Imazethypyr), Phenoxy (2,4-D, 2,4,5-T, 2,4,5-TP), Phosphinic (Glyphosate), Thiocarbamate (Butylate, EPTC), Triazine (Atrazine, Cyanazine, Metribuzin), Urea (Chlorimuron-ethyl), other herbicides (Paraquat, Petroleum oil) <u>Insecticides:</u> Carbamate (Aldicarb, Carbaryl, Carbofuran), Organochlorine (Aldrin, Chlordane, DDT, Dieldrin, Heptachlor, Lindane, Toxaphene), Oganophosphate (Chlorpyrifos, Coumaphos, Diazinon, Dichlorvos, Fonofos, Malathion, Parathion, Phorate, Terbufos), Pyrethroid (Permethrin crops and animals) <u>Fungicides:</u> Benomyl, Captan, Chlorothalonil, Maneb/Mancozeb, Metalaxyl <u>Fumigants:</u> Aluminum phosphide, Carbon disulfide/Carbon tetrachloride, Ethylene dibromide, Methyl bromide
Silver et al. [130]	Cancer incidence and Metolachlor use in the Agricultural Health Study: An update	2015	Cohort (AHS)	Cancer	Cancer registry files in Iowa and North Carolina	USA	ND	1993-2011	49,616 pesticide applicators (26,505 reported using Metolachlor) (5,701 incident cancers)	Metolachlor and Atrazine, Alachlor, Imazethapyr, Trifluralin, Dicamba
Starks et al. [131]	Peripheral Nervous System Function and Organophosphate Pesticide Use among Licensed Pesticide Applicators in the Agricultural Health Study	2012	Cohort (AHS)	Neurotoxicity	Neurological testing	USA	ND	1993-1997	701 male applicators	<u>Insecticides:</u> Organophosphates (Acephate, Chlorpyrifos, Coumaphos, Diazinon, Dichlorvos, Dimethoate, Disulfoton, Ethoprop, Fonofos, Malathion, Parathion, Phorate, Phosmet, Tebupirimfos, Terbufos, Tetrachlorvinphos) Carbamates (Aldicarb, Benomyl, Carbaryl,

										Carbofuran)<br>HPEEs
Starling et al. [132]	Pesticide use and incident diabetes among wives of farmers in the Agricultural Health Study	2014	Cohort (AHS)	Endocrine disruption (Diabete)	self-administrated questionnaires or telephone interviews and telephone interviews for follow-up	USA	ND	1993-1997	13,637 farmers' wives who reported ever personally mixing or applying pesticides at enrollment applicators and their spouses	45 active ingredients:<br>Herbicides: Benzoic (Dicamba), Chloroacetanilide (Alachlor, Metolachlor), Dinitroaniline (Pendimethalin, Trifluralin), Imidazolinone (Imazethypyr), Phenoxy (2,4-D, 2,4,5-T, 2,4,5-TP), Phosphinic (Glyphosate), Thiocarbamate (Butylate, EPTC), Triazine (Atrazine, Cyanazine, Metribuzin), Urea (Chlorimuron-ethyl), other herbicides (Paraquat, Petroleum oil)<br>Insecticides: Carbamate (Aldicarb, Carbaryl, Carbofuran), Organochlorine (Aldrin, Chlordane, DDT, Dieldrin, Heptachlor, Lindane, Toxaphene), Oganophosphate (Chlorpyrifos, Coumaphos, Diazinon, Dichlorvos, Fonofos, Malathion, Parathion, Phorate, Terbufos), Pyrethroid (Permethrin crops and animals)<br>Fungicides: Benomyl, Captan, Chlorothalonil, Maneb, Metalaxyl<br>Fumigants: Carbon disulfide/Carbon tetrachloride, Ethylene dibromide, Methyl bromide
Talibov et al. [133]	Colorectal cancer among farmers in the AGRICAN cohort study	2022	Cohort (AGRICAN)	Cancer	Cancer registries	France	13 types of crops + 5 types of livestock	2005-2015	2609 cases of colorectal cancer	Insecticides use on livestock<br>Insecticide treatment in horse farming<br>Pesticide treatment in sunflower, fruit growing, tobacco
Tual et al. [134]	Occupational exposure to pesticides and multiple myeloma in the AGRICAN cohort	2019	Cohort (AGRICAN)	Cancer	Cancer registries	France	13 types of crops + 5 types of livestock	2005-2013	155,192 women and men active and retired in agriculture-related	Insecticides use on animals (DDT, lindane, carbaryl, carbaryl, permethrin, coumaphos)<br>Disinfectants in barns (quaternary ammonium, phenol derivatives, aldehydes including formaldehyde)

									activities (269 incident cases of multiple myeloma)	Pesticides use on corn (DDT, lindane, carbaryl, 2-4-D, captan, atrazine, dinoterb)
van Bemmel et al. [135]	S-Ethyl-N,N-dipropylthiocarbamate exposure and cancer incidence among male pesticide applicators in the Agricultural Health Study: A prospective cohort	2008	Cohort (AHS)	Cancer	Cancer registry files in Iowa and North Carolina	USA	Only analysis of Field corn production	1993-2004	48,378 pesticide applicators (9,878 reported using EPTC) (470 incident cancer cases)	EPTC

Table S2. Overall risk of bias assessment following the Joanna Briggs Institute (JBI) checklist for prevalence studies.

Authors	Publication year	Q1*	Q2*	Q3*	Q4*	Q5*	Q6*	Q7*	Q8*	Q9*	Somme	Classification
Alavanja et al. [69]	2003	1	1	1	1	1	1	1	1	1	9	low
Alavanja et al. [70]	2004	1	1	1	0	0	1	1	1	0	6	high
Alavanja et al. [71]	2014	1	1	1	1	1	1	1	1	1	9	low
Andreotti et al. [72]	2009	1	1	1	0	1	1	1	1	0	7	moderate
Andreotti et al. [73]	2018	1	1	1	1	1	1	1	1	0	8	moderate
Andreotti et al. [16]	2020	1	1	1	0	1	1	1	1	1	8	moderate
Band et al. [74]	2011	1	1	0	1	1	1	1	1	1	8	moderate
Barry et al. [75]	2012	1	1	1	1	1	1	1	1	0	8	moderate
Beane Free-man et al. [76]	2005	1	1	1	1	0	1	1	1	1	8	moderate
Bonner et al. [77]	2007	1	1	1	1	1	1	1	1	0	8	moderate
Bonner et al. [78]	2010	1	1	0	1	1	1	1	1	0	7	moderate
Bonner et al. [79]	2017	1	1	1	1	0	1	1	1	1	8	moderate
Boulanger et al. [62]	2017	1	1	1	1	1	1	0	1	0	7	moderate
Boulanger et al. [80]	2018	1	1	0	1	1	1	1	1	0	7	moderate
Chiu et al. [81]	2006	1	1	1	0	1	1	0	1	0	6	high

Christensen et al. [82]	2010	1	1	1	1	1	1	1	1	0	8	moderate
De Roos et al. [83]	2005	1	1	1	0	1	1	1	1	0	7	moderate
Delancey et al. [84]	2009	1	1	1	1	1	1	1	1	0	8	moderate
Dennis et al. [85]	2010	1	1	1	1	1	1	1	1	0	8	moderate
Engel et al. [86]	2005	1	1	1	0	0	1	1	1	0	6	high
Goldner et al. [87]	2013	1	1	1	1	0	0	1	1	1	7	moderate
Greenburg et al. [88]	2008	1	1	1	1	0	1	1	1	1	8	moderate
Hofmann et al. [89]	2021	1	1	1	1	1	1	1	0	1	8	moderate
Hou et al. [90]	2006	1	1	1	1	1	1	1	1	0	8	moderate
Ji et al. [91]	2001	0	1	1	0	1	1	1	1	0	6	high
Jones et al. [92]	2015	1	1	1	1	1	1	0	1	0	7	moderate
Kamel et al. [93]	2007	1	1	1	0	0	0	0	1	0	4	high
Kang et al. [94]	2008	1	1	1	1	0	1	1	1	0	7	moderate
Koutros et al. [95]	2008	1	1	1	1	0	1	1	1	0	7	moderate
Koutros et al. [96]	2009	1	1	1	1	1	1	1	1	0	8	moderate
Koutros et al. [98]	2016	1	1	1	0	1	1	1	1	1	8	moderate
Koutros et al. [97]	2013	1	1	1	1	1	1	1	1	0	8	moderate
Lee et al. [99]	2004	1	1	1	1	1	1	1	1	0	8	moderate

Lee et al. [100]	2004	1	1	1	1	0	1	1	1	1	8	moderate
Lee et al. [101]	2007	1	1	0	1	1	1	1	1	0	7	moderate
Lemarchand et al. [102]	2016	1	1	1	1	1	1	0	1	0	7	moderate
Lerro et al. [103]	2015	1	1	1	1	1	1	1	1	0	8	moderate
Lerro et al. [104]	2020	1	1	1	1	0	1	1	1	0	7	moderate
Lerro et al. [105]	2021	1	1	1	1	1	1	1	1	0	8	moderate
Lerro et al. [106]	2018	1	1	1	1	0	1	1	1	0	7	moderate
Lynch et al. [107]	2006	1	1	0	1	0	1	1	1	1	7	moderate
Lynch et al. [108]	2009	1	1	0	1	0	1	1	1	1	7	moderate
Mahajan et al. [109]	2006	1	1	0	1	1	1	1	1	0	7	moderate
Mahajan et al. [110]	2006	1	1	0	1	1	1	1	1	0	7	moderate
Mahajan et al. [111]	2007	1	1	0	1	1	1	1	1	0	7	moderate
Mills and Yang [112]	2003	1	1	0	1	1	1	1	1	1	8	moderate
Mills et al. [113]	2005	1	1	0	0	1	1	1	1	0	6	high
Montgomery et al. [114]	2008	1	1	1	1	0	0	0	1	0	5	high
Mozzachio et al. [115]	2008	1	1	0	1	1	1	1	1	1	8	moderate

Narayan et al. [116]	2017	1	1	0	0	1	0	1	1	0	5	high
Pardo et al. [117]	2020	1	1	1	0	1	1	1	1	1	8	moderate
Park et al. [118]	2009	1	1	0	1	0	1	1	1	0	6	high
Park et al. [119]	2019	1	1	0	1	1	0	0	0	0	4	high
Piel et al. [64]	2017	1	1	0	1	1	1	1	1	0	7	moderate
Piel et al. [120]	2019	1	1	1	1	1	1	1	1	0	8	moderate
Piel et al. [121]	2019	1	1	0	1	1	1	1	1	1	8	moderate
Pouchieu et al. [65]	2018	1	1	0	1	1	1	0	1	0	6	high
Purdue et al. [122]	2007	1	1	1	1	0	1	1	1	0	7	moderate
Renier et al. [123]	2022	1	1	1	1	1	1	0	1	1	8	moderate
Rusiecki et al. [124]	2004	1	1	1	1	1	1	1	1	1	9	low
Rusiecki et al. [125]	2006	1	1	1	1	1	1	1	1	1	9	low
Rusiecki et al. [126]	2009	1	1	1	1	0	1	1	1	0	7	moderate
Samanic et al. [127]	2006	1	1	1	1	0	1	1	1	1	8	moderate
Shrestha et al. [128]	2020	1	1	1	0	1	0	1	1	0	6	high
Shrestha et al. [129]	2018	1	1	1	1	1	0	1	1	0	7	moderate
Silver et al. [130]	2015	1	1	1	1	0	1	1	1	1	8	moderate

Starks et al. [131]	2012	1	1	1	1	0	1	1	1	0	7	moderate
Starling et al. [132]	2014	1	1	1	1	0	0	0	1	0	5	high
Talibov et al. [133]	2022	1	1	1	1	1	1	0	1	1	8	moderate
Tual et al. [134]	2019	1	1	0	1	1	1	1	1	1	8	moderate
van Bemmel et al. [135]	2008	1	1	1	1	0	1	1	1	0	7	moderate

*Q1. Was the sample frame appropriate to address the target population?
Q2. Were study participants sampled in an appropriate way?
Q3. Was the sample size adequate?
Q4. Were the study subjects and the setting described in detail?
Q5. Was the data analysis conducted with sufficient coverage of the identified sample?
Q6. Were valid methods used for the identification of the condition?
Q7. Was the condition measured in a standard, reliable way for all participants?
Q8. Was there appropriate statistical analysis?
Q9. Was the response rate adequate, and if not was the low response rate managed appropriately?
